# Osteoclast-expanded super-charged NK-cells preferentially select and expand CD8+ T cells

**DOI:** 10.1038/s41598-020-76702-1

**Published:** 2020-11-23

**Authors:** Kawaljit Kaur, Meng-Wei Ko, Nick Ohanian, Jessica Cook, Anahid Jewett

**Affiliations:** 1 Division of Oral Biology and Oral Medicine, School of Dentistry and Medicine, Los Angeles, CA USA; 2 The Jane and Jerry Weintraub Center for Reconstructive Biotechnology, UCLA School of Dentistry and Medicine, 10833 Le Conte Ave, Los Angeles, CA 90095 USA; 3 The Jonsson Comprehensive Cancer Center, UCLA, Los Angeles, CA USA

**Keywords:** Cancer, Immunology

## Abstract

Osteoclasts (OCs) and much less dendritic cells (DCs) induce significant expansion and functional activation of NK cells, and furthermore, the OC-expanded NK cells preferentially increase the expansion and activation of CD8+ T cells by targeting CD4+ T cells. When autologous OCs were used to expand patient NK cells much lower percentages of expanded CD8+ T cells, decreased numbers of expanded NK cells and decreased functions of NK cells could be observed, and the addition of allogeneic healthy OCs increased the patients’ NK function. Mechanistically, OC-expanded NK cells were found to lyse CD4+ T cells but not CD8+ T cells suggesting potential selection of CD8+ T cells before their expansion by OC activated NK cells. In agreement, Increased IFN-γ secretion, and NK cell-mediated cytotoxicity and higher percentages of CD8+ T cells, in various tissue compartments of oral tumor-bearing hu-BLT mice in response to immunotherapy by OC-expanded NK cells were observed. Thus, our results indicate an important relationship between NK and CD8+ T cells.

## Introduction

Tumor cells are known to decrease the numbers of NK and CD8+ T cells as well as suppressing their function^1–5^. Similar profiles of decrease in NK and CD8+ T cells have been seen in patients suffering from COVID-19^6,7^. The interconnectedness of these two important cytotoxic cells has not been clearly delineated previously even though correlations were seen in increased percentages of NK and CD8+ T cells in peripheral blood and in bone marrow (BM) of cancer patients in the presence of decrease in CD4+ T cells^8–12^. It is of particular interest to note that such profile is largely seen in the BM of multiple myeloma (MM) patients where osteoclasts (OCs) are found. OCs are largely known for their influence on the turnover rate of the bone. However, in recent years the role of OCs in the modulation of different immune effectors, in particular NK cells has received great attention^13–15^. Therefore, even though the classical function of OCs were long regarded as influencing the architecture of the bone, recent reports from our laboratory and those of the others have bestowed a well-deserved status to OCs as immunomodulatory effectors which their function go beyond their well-known role in bone remodeling^13–17^.

NK and T cells comprise a large portion of the lymphocyte population in peripheral blood mononuclear cells (PBMCs). NK cells are mainly known as the effectors of innate immunity due to their lack of antigen-specificity; however, this notion has been challenged in recent years^18–20^. Decreased peripheral blood lymphocyte infiltration and lower cytotoxic activity, particularly in NK cells, result in poor prognosis in cancer patients^21–26^. However, cancer-mediated immune suppression remains a complex and multifactorial problem. Various studies have shown that several factors in the tumor microenvironment suppress NK cells by downregulating NK cell surface receptors^27–34^. Studies have shown that NK cells can activate and induce the proliferation of T cells through direct cell–cell contact^35,36^; IL-2–activated NK cells can also directly induce the maturation of DCs and enhance their ability to stimulate allogeneic naive CD4+ T cells^37^. Several other studies have demonstrated the immunoregulatory roles of NK cells which play in killing chronically activated leukocytes^38–41^, and eliminating activated autologous CD4+ T cells^41–43^.

CD4+ and CD8+ T cells mediate the adaptive cellular immunity, which closely collaborate with the innate immune system^44,45^. CD8+ T cells spearhead the cellular immune responses that protect against tumors^46^. High levels of tumor-infiltrating CD8+ T cells are associated with complete responses to standard chemotherapeutic regimens^47^, and the presence of CD8+ memory T cells is associated with cancer patients’ survival^48,49^. Increased T regulatory (Treg) cell infiltration into solid tumors is negatively associated with overall survival in cancer patients^50^.

NK and CD8+ T cell-based immunotherapies are among the leading standards in cancer therapeutics^51,52^. NK immunotherapies have been limited due to the low numbers of these cells in the peripheral blood and our inability to expand large numbers of functional NK cells with extended survival in patients. To overcome this problem, we previously introduced a novel NK expansion methodology using a combination of OCs and sonicated probiotic bacteria (sAJ2) to generate activated super-charged NK cells with the potential to kill and differentiate cancer stem cells (CSCs)^15^. Later in the expansion process of NK cells by OCs we have also observed that this methodology led to the expansion of a very small population of contaminating T cells which were initially not detectable^15^. However, after many rounds of expansion a small population of CD8+ T cells was expanding in NK cultures which coincided with the lower ability of NK cells to lyse tumors^15^. Previous studies have also shown that NK cells can accelerate CD8+ T cell responses against viral infections, such as those caused by cytomegaloviruses^53,54^. We have also uncovered the differences in the dynamics of NK cell expansion in healthy individuals and cancer patients^15^.

In this study we sought to determine how NK and T cells differ in their numbers and responses in cancer patients and in healthy individuals using our novel methodology to expand NK cells. In addition, we observed that OC-activated super-charged NK cells specifically expand CD8+ T cells, whereas DC-activated NK cells demonstrate a preference for the CD4+ T cell expansion. Our *in-vitro* study revealed how supercharged NK cells might affect the balance of T cell subsets, cytokine secretions, and cytotoxic activity of immune cells in various tissue compartments of the healthy and cancer-bearing hu-BLT mice. Finally, we demonstrated that super-charged NK cells lyse activated CD4+ T and not CD8+ T cells, thus selecting and preferentially expanding CD8+ T cells.

## Results

### Decreased numbers and suppression of cytotoxicity and secretion of IFN-γ by NK cells in cancer patients

The numbers of PBMCs were significantly lower in the peripheral blood of cancer patients when compared to healthy individuals when identical amounts of blood was used to isolate PBMCs (Fig. S1A). Higher percentages of CD16+ CD56+ , CD14+ , and CD11b+ , and lower percentages of CD3+ and CD19+ cells were obtained within PBMCs of cancer patients when compared to healthy individuals (Fig. S1B). Cancer patients’ NK cells secreted significantly lower amounts of IFN-γ (Fig. S1C and S1E) and mediated lower cytotoxicity (Fig. S1D). In addition to IFN-γ, cancer patients’ NK cells also secreted significantly lower levels of other cytokines (Fig. S1E). Decreased levels of cytokines were also seen in the sera of cancer patients when compared to those of healthy individuals (Fig. S1F). These findings indicated that cancer patients’ peripheral blood contains fewer PBMCs and exhibit higher proportions of NK cells with substantially lower NK cell function in comparison to those of healthy individuals.

### Allogeneic OC-mediated expansion, and augmented function of NK cells from cancer patients is greatly suppressed when compared to those of healthy individuals

**Figure 1:**
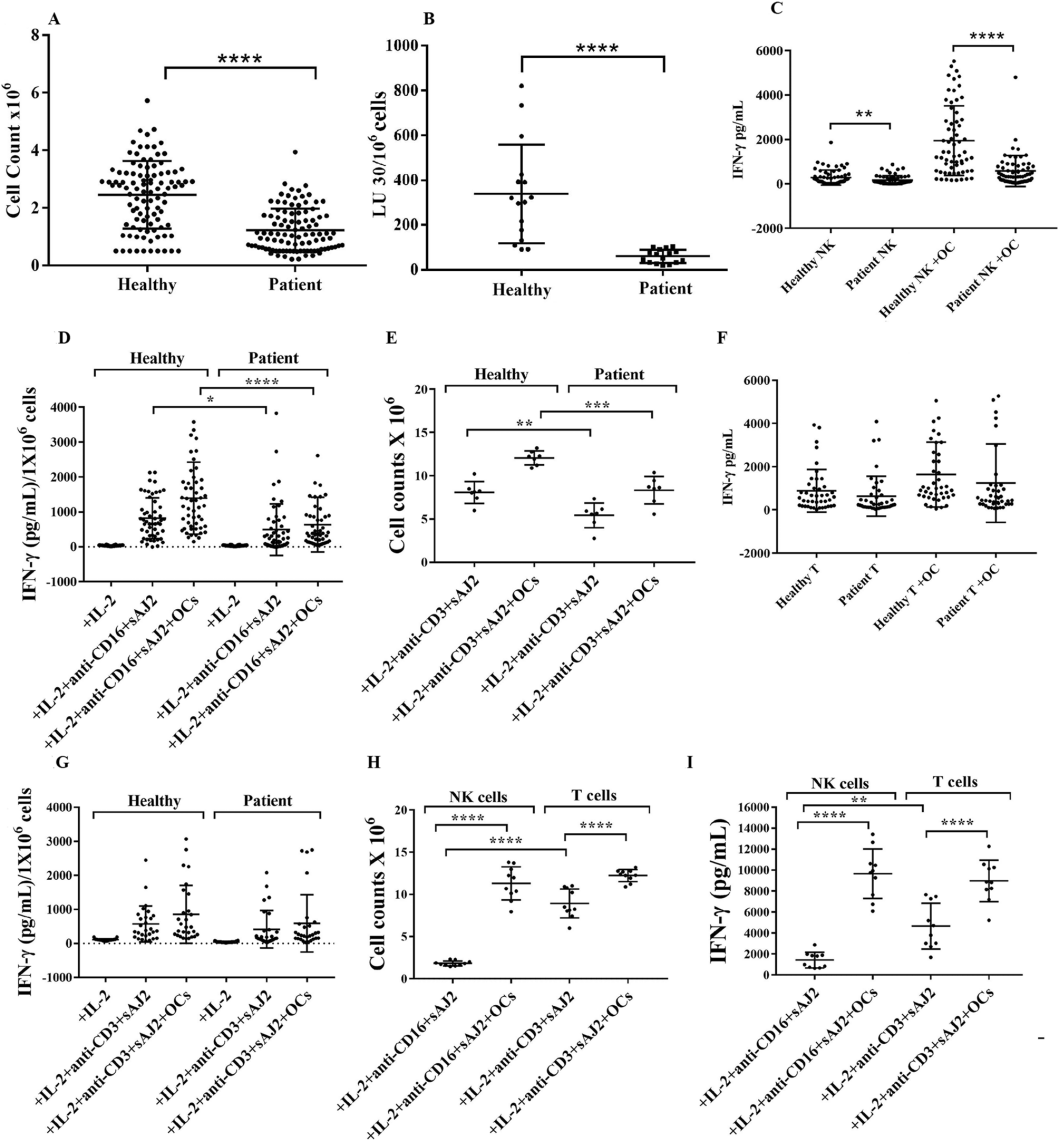
OC-expanded NK cells from cancer patients have much lower capacity to expand, mediate cytotoxicity, and secrete IFN-γ. OCs were generated as described in Materials and Methods. NK cells (1 × 10^6^ cells/ml) from healthy individuals and cancer patients were treated with a combination of IL-2 (1000 U/ml) and anti-CD16mAb (3 µg/ml) for 18 h before they were cultured with the healthy individuals’ OCs and sAJ2 at a ratio of 1:2:4 (OCs:NK:sAJ2). On days 6, 9, 12, and 15 of co-culture, the numbers of lymphocytes were counted using microscopy (n = 70) **(A)**. NK cells were treated and cultured as described in Fig. 1A. Cytotoxicity of day 15 cultured NK cells was determined using standard 4-h ^51^Cr release assay against OSCSCs. The Lytic units (LU) 30/10^6^ cells were determined using the inverse number of NK cells required to lyse 30% of OSCSCs × 100 (n = 16) **(B)**. NK cells were treated and cultured as described in Fig. 1A. On days 6, 9, 12, and 15, supernatants were harvested from the co-cultures to determine IFN-γ secretion using single ELISA (n = 63) **(C)**. The amounts of IFN-γ secretion shown in Fig. 1C were determined based on 1 × 10^6^ cells (n = 63) **(D)**. T cells (1 × 10^6^ cells/ml) from healthy individuals and cancer patients were treated with a combination of IL-2 (100 U/ml) and anti-CD3 (1 µg/ml)/CD28mAb (3 µg/ml) for 18 h before they were co-cultured with healthy individuals’ OCs and sAJ2 at a ratio of 1:2:4 (OCs:T:sAJ2). On days 6, 9, 12, and 15, the T cells were counted using microscopy; the cumulative cell counts from day 0 to day 15 are displayed in the figure (n = 7) **(E)**. T cells were treated and cultured as described in Fig. 1E. The supernatants were harvested on days 6, 9, 12, and 15, and the levels of IFN-γ secretion were determined using single ELISA (n = 42) **(F)**. Amounts of IFN-γ secretion shown in Fig. 1F were assessed based on 1 × 10^6^ cells (n = 42) **(G)**. NK cells and T cells were treated and cultured as described in Fig. 1A and Fig. 1E respectively. The cells were counted using microscopy on days 6, 9, 12, and 15; the cumulative cell counts from day 0 to day 15 are displayed in the figure (n = 10) **(H)**. NK and T cells were treated and cultured as described in Fig. 1A and Fig. 1E respectively. The supernatants were then harvested on days 6, 9, 12, and 15 of the co-cultures and the amounts of IFN-γ secretion were determined using single ELISA; the cumulative levels of IFN-γ secretion from day 0 to day 15 are displayed in the figure (n = 10) **(I)**.

**Figure 2:**
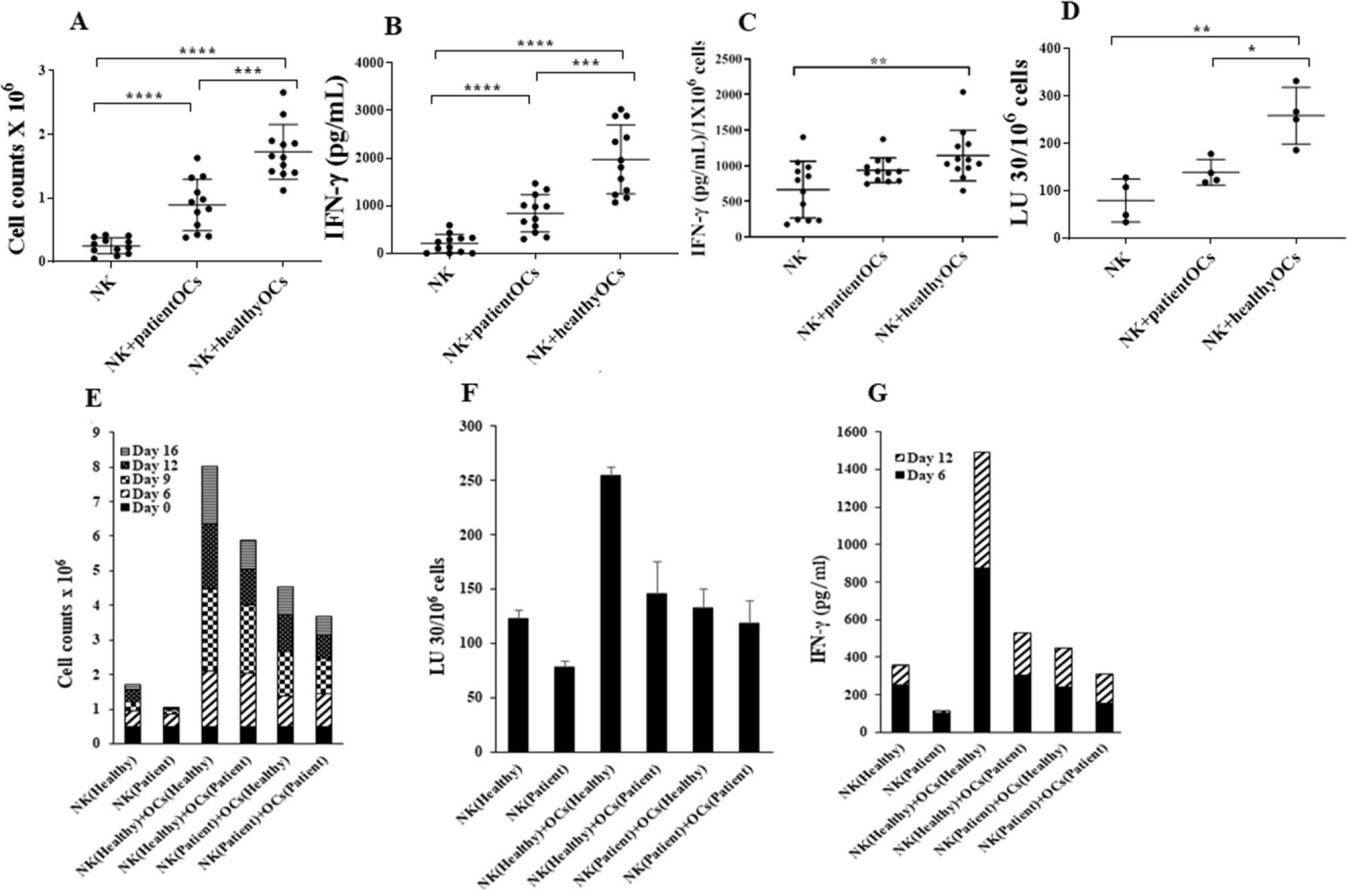
Unlike those from healthy individuals, OCs from cancer patients induced decreased cell expansion, IFN-γ secretion and cytotoxicity in allogeneic NK cells obtained from healthy individuals. NK cells (1 × 10^6^ cells/ml) from healthy individuals were treated with the combination of IL-2 (1000 U/ml) and anti-CD16mAb (3 µg/ml) for 18 h before they were cultured alone or were co-cultured with either healthy individuals’ OCs or cancer patients’ OCs in the presence of sAJ2 at a ratio of 1:2:4 (OCs:NK:sAJ2). On days 6, 9, 12, 15, 18 and 22 of co-culture, the numbers of NK cells were counted using microscopy (n = 12) **(A)**. NK cells were treated and co-cultured as described in Fig. 2A. On days 6, 9, 12, 15, 18 and 22, supernatants were harvested from the co-cultures to determine IFN-γ secretion using single ELISA (n = 12) **(B)**. The amounts of IFN-γ secretion shown in Fig. 2B were assessed based on 1 × 10^6^ cells (n = 12) **(C)**. NK cells were treated and co-cultured as described in Fig. 2A. Cytotoxicity of days 9 and 15 cultured NK cells were determined using a standard 4-h ^51^Cr release assay against OSCSCs. LU 30/10^6^ cells were determined as described in Fig. 1B (n = 4) **(D)**. NK cells (1 × 10^6^ cells/ml) from healthy individuals and cancer patients were treated with the combination of IL-2 (1000 U/ml) and anti-CD16mAb (3 µg/ml) for 18 h before they were cultured alone, or with autologous OCs in the presence of sAJ2 at a ratio of 1:2:4 (OCs:NK:sAJ2). On days 6, 9, 12, and 16 of co-culture, the numbers of NK cells were counted using microscopy **(E)**. NK cells were treated and co-cultured as described in Fig. 2E. Cytotoxicity of days 9 and 15 cultured NK cells were determined using a standard 4-h ^51^Cr release assay against OSCSCs. LU 30/10^6^ cells were determined as described in Fig. 1B (n = 4) **(F)**. NK cells were treated and co-cultured as described in Fig. 2E. On days 6 and 12, supernatants were harvested from the co-cultures to determine IFN-γ secretion using single ELISA **(G)**.

To determine the extent of NK and T cell expansion and function, we expanded NK and T cells of cancer patients and healthy individuals using our expansion strategy as previously described^15^. Cancer patients’ NK cells showed significantly decreased levels of expansion (Figs. 1A, 2E), and expanded NK cells exhibited significantly lower cytotoxicity (Figs. 1B, 2F), and IFN-γ secretion (Figs. 1C, D, S2 and 2G) when compared to those of healthy individuals. Cancer patients’ T cells exhibited similar decreases in expansion rate (Figs. 1E and S3A) and IFN-γ secretion (Figs. 1F–G and S3B-S3E).

To assess whether NK and T cells exhibit distinct expansion profiles, we cultured NK and T cells of healthy individuals in the presence and absence of OCs and found that T cells expanded faster than NK cells in the absence of OCs (Figs. 1H and S3F). However, OCs induced 2.6–4.5 fold and 1.2–1.6 fold expansion in NK and T cells, respectively, when compared to those cultured in the absence of OCs (Figs. 1H and S3F). These results indicated that OCs induce higher expansion of NK cells when compared to T cells. T cells secreted significantly higher IFN-γ secretion in the absence of OCs while IFN-γ secretion in both NK and T cells exhibited comparable increases in the presence of OCs (Fig. 1I).

### Cancer patients’ OCs induced lower cell expansion, IFN-γ secretion and cytotoxicity in NK cells when compared to healthy individuals’ OCs

To investigate the function of patients’ OCs, we cultured the healthy individuals’ NK cells with either autologous OCs or with patients’ OCs (allogeneic). Patients’ OCs were less capable of inducing NK cell expansion (Fig. 2A,E), IFN-γ secretion (Fig. 2B,C,G), and NK cytotoxicity (Fig. 2C,F). We next assessed the function of patient NK cells in the context of autologous OCs. Severe decreases in NK cell expansion, IFN-γ secretion, and cytotoxicity were observed when patients’ NK cells were cultured with autologous OCs (Fig. 2E–G). These results indicated severe functional defects in both NK cells and OCs of cancer patients.

### OC-induced T cell mediated expansion increased CD8+ T cells moderately when compared to OC-induced NK cell mediated expansion of CD8+ T cells

**Figure 3:**
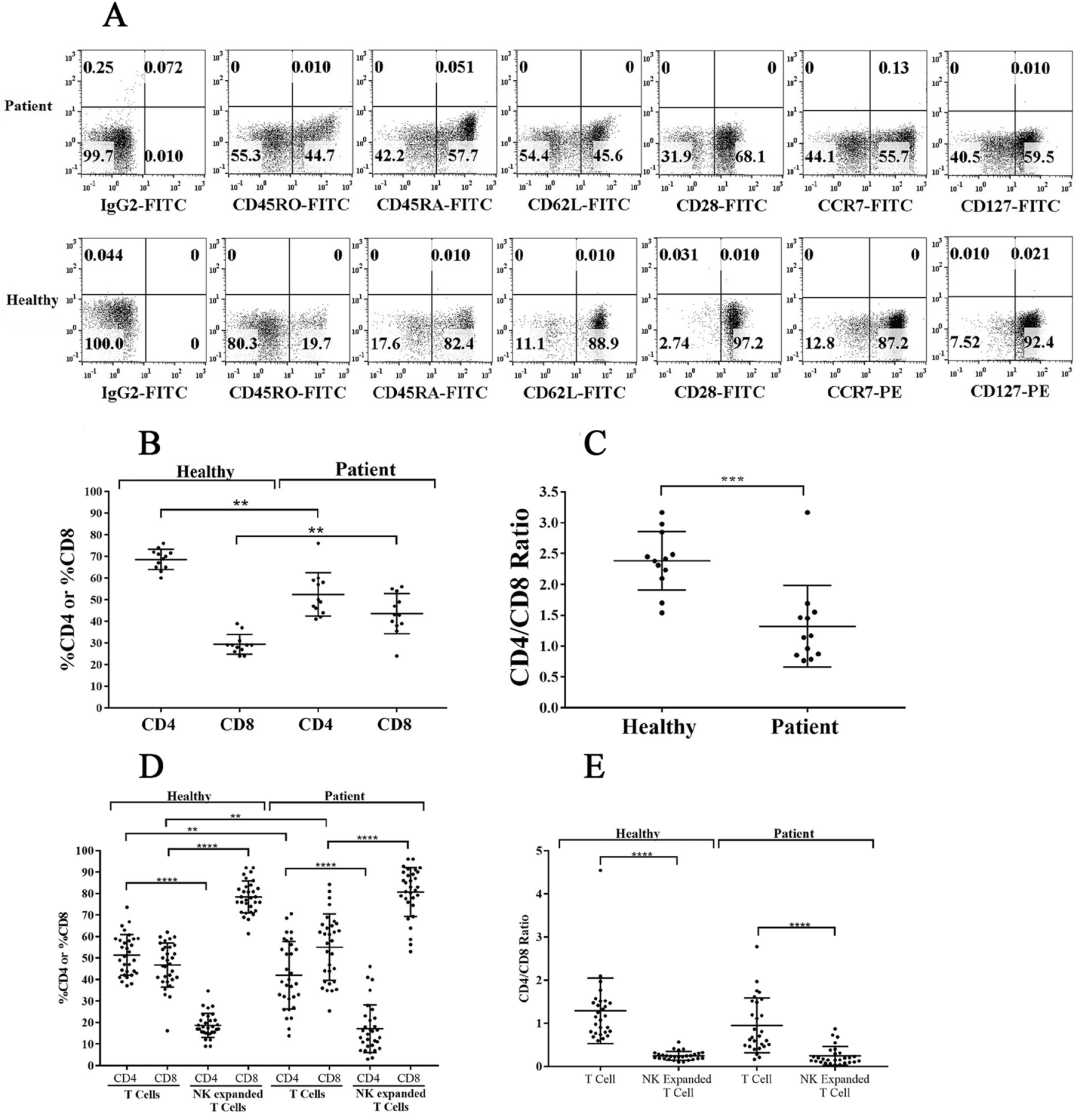
T cells from cancer patients exhibit lower CD4 + /CD8 + T cell ratio both in peripheral blood and after expansion. T cells purified from PBMCs of healthy individuals and cancer patients were analyzed for the surface expression of CD45RO, CD45RA, CD62L, CD28, CCR7, and CD127 using flow cytometry. IgG isotype control was used to assess non-specific binding. One of 12 representative experiments is shown in the figure **(A)**. PBMCs isolated from the peripheral blood of healthy individuals and cancer patients were used to determine the surface expression of CD4 and CD8 using flow cytometry, and the percentages of CD4 + and CD8 + T cells were determined within CD3 + populations (n = 12) **(B)**. The ratio of CD4:CD8 is shown in the figure (n = 12) **(C)**. NK and T cells of healthy individuals and cancer patients were treated and co-cultured as described in Fig. 1A and Fig. 1E respectively. On days 6, 9, 12, and 15 of co-culture, the surface expression of CD4 and CD8 were analyzed by flow cytometry to obtain the percentages of CD4 + and CD8 + T cells within CD3 + populations (n = 28) **(D)**. The ratio of CD4:CD8 is shown in the figure (n = 28) **(E)**.

We next analyzed the surface phenotype of memory and naïve subpopulations of T cells, and observed increase in CD45RO+ cells (activated T cells) and decrease in CD45RA+ cells (naïve T cells) on cancer patients’ T cells (Fig. 3A). We also noted reduced surface expressions of CD62L, CD28, CCR7, and CD127 on cancer patients’ T cells (Fig. 3A). Moreover, percentages of CD4+ T cells were decreased with corresponding increase in the percentages of CD8+ T cells in cancer patients’ PBMCs (Fig. 3B). Accordingly, we observed decreased CD4+ /CD8+ T cell ratios in cancer patients’ PBMCs, suggesting an overall increase in the CD8+ T cell subset in cancer patients when compared to healthy individuals (Fig. 3C).

We next cultured NK cells in the presence of healthy allogeneic OCs and determined the fractions of expanded CD4+ and CD8+ T cells within the expanded NK cells. No detectable T cells could be seen initially after NK cell purifications, however, after several rounds of NK cell expansions we were able to detect T cell expansion within the NK cells. The expanded T cells were primarily CD8+ T cells with no or very low levels of CD4+ T cells in cultures of expanded NK cells from both healthy individuals and cancer patients (Fig. 3D), and the relative CD4+/CD8+ T cell ratios remained similar between cancer patients and healthy individuals (Fig. 3E). However, it should be noted that the patients have higher percentages of CD8+ T cells than CD4+ T cells in their PBMCs when compared to those of healthy individuals (Fig. 3B–E).

When purified T cells were cultured with allogeneic healthy OCs, cancer patients but not healthy individuals exhibited higher percentages of CD8+ T cells with lower CD4+ /CD8+ T cell ratios since the levels of CD8+ T cells were constitutively higher in cancer patients PBMCs in the absence of expansion (Fig. 3D,E). Also, it should be noted that for the sake of comparison we chose to activate T cells by IL-2 and anti-CD3/CD28 signaling since NK cells were activated by IL-2 and anti-CD16 mAbs before they were cultured with OCs. Thus, NK and T cells were pre-activated before their culture with OCs. We observed much higher percentages and numbers of CD8+ T cell expansion with no or very low remaining CD4+ T cells in OC-expanded NK cells, whereas OC-induced T cells expanded CD8+ T cells moderately with significant percentage of CD4+ T cells still remaining in the culture (Fig. 3D,E).

### Increased NK numbers and NK-mediated cytotoxicity by OC-expansion in comparison to DC-expansion; OCs preferentially expand CD8+ T cells whereas DCs preferentially expand CD4+ T cells in NK cells cultures

**Figure 4:**
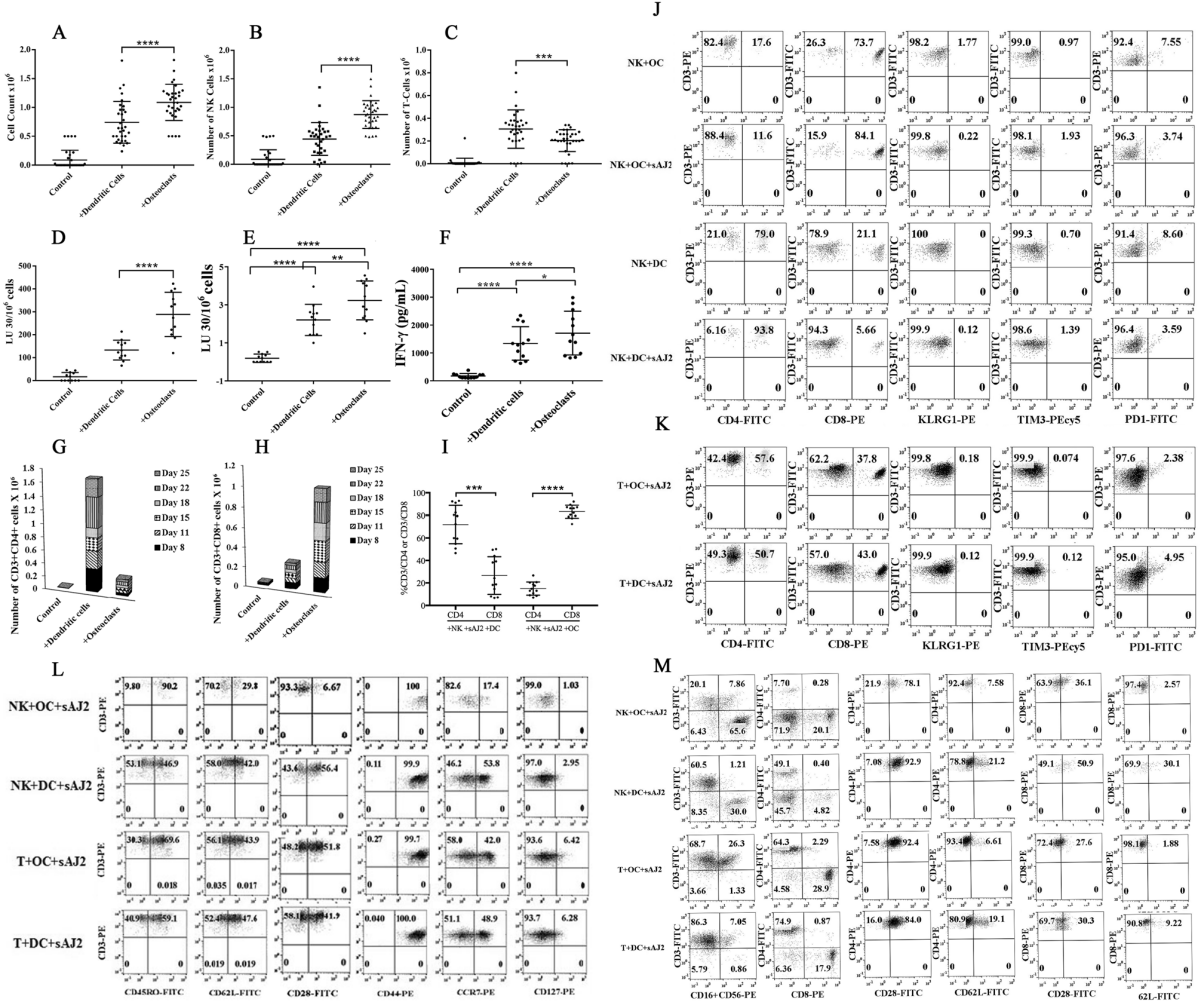
OC-expanded NK cells induced CD8 + T cell expansion whereas DC-expanded NK cells promote CD4 + T cell expansion. OCs and DCs were generated as described in Materials and Methods. NK cells from healthy individuals (1 × 10^6^ cells/ml) were treated with a combination of IL-2 (1000 U/ml) and anti-CD16mAb (3 µg/ml) for 18 h before they were co-cultured with autologous DCs or OCs in the presence of sAJ2 at 1:2:4 ratios (DCs or OCs:NK:sAJ2). The expanding cells were counted on days 8, 11, 15, and 18 using microscopy (n = 30) **(A).** NK cells were co-cultured with OCs or DCs as described in Fig. 4A, and the surface expressions of CD3, CD16, and CD56 were analyzed on days 8, 11, 15, and 18 using flow cytometry. The numbers of NK cells and T cells were determined using the percentages of CD16 + and CD3 + cells, respectively, within the total cells in Fig. 4A (n = 30) **(B, C)**. NK cells were co-cultured with OCs or DCs as described in Fig. 4A and cytotoxicity of day 15 expanded cells was determined using a standard 4-h ^51^Cr release assay against OSCSCs. LU 30/10^6^ cells were determined using the method described in Fig. 1B (n = 12) **(D)**. NK cells were co-cultured with OCs or DCs as described in Fig. 4A, and the surface expressions of CD16 were analyzed on day 15 using flow cytometry. The levels of the cytotoxicity was determined based on 1% of CD16 + NK cells (n = 12) **(E)**. NK cells were co-cultured with OCs or DCs as described in Fig. 4A; the supernatants were harvested on days 8, 11, 15, and 18 of the co-cultures, and the amounts of IFN-γ secretion were determined using single ELISA (n = 12) **(F)**. NK cells were co-cultured with OCs or DCs as described in Fig. 4A. On days 8, 11, 15, 18, 22 and 25 of the co-cultures the surface expressions of CD3 + CD4 + and CD3 + CD8 + T cells were determined using flow cytometry, and the percentages were used to determine the total numbers of CD3 + CD4 + and CD3 + CD8 + cells within the total cells (n = 12) **(G, H)**. NK cells were co-cultured with OCs or DCs as described in Fig. 4A, and the surface expressions of CD3, CD4, and CD8 were analyzed on days 8, 11, 15, and 18 using flow cytometry. Percentages of CD4 + and CD8 + T cells within the CD3 + populations are shown in this figure (n = 12) **(I)**. NK cells were co-cultured with OCs or DCs as described in Fig. 4A and the surface expressions of CD4, CD8, KLRG1, TIM3, and PD-1 were analyzed within CD3 + cells on day 27 of the co-cultures using flow cytometry (n = 8) **(J).** T cells (1 × 10^6^ cells/ml) from healthy individuals were treated with a combination of IL-2 (100 U/ml) and anti-CD3 (1 µg/ml)/CD28mAb (3 µg/ml) for 18 h before they were co-cultured with autologous DCs or OCs in the presence of sAJ2 at 1:2:4 ratios (DCs or OCs:T:sAJ2). Surface expressions of CD4, CD8, KLRG1, TIM3, and PD-1 were analyzed within CD3 + cells on day 27 of the co-culture using flow cytometry (n = 8) **(K)**. NK and T cells were co-cultured with OCs or DCs as described in Fig. 4A and Fig. 4 K, respectively. Surface expressions of CD45RO, CD62L, CD28, CD44, CCR7, and CD127 were analyzed within CD3 + cells on day 12 of the co-culture using flow cytometry (n = 8) **(L)**. NK and T cells were co-cultured with OCs or DCs as described in Fig. 4A and Fig. 4 K, respectively and the surface expressions of CD3, CD16, CD56, CD4, CD8, CD28, and CD62L were analyzed on day 12 of the co-culture using flow cytometry (n = 8) **(M)**.

To assess whether the activation of NK cells by OCs vs. DCs differentially affects expansion profile and function, we cultured NK cells from healthy individuals either alone, with OCs, or with DCs. Significantly higher cell counts were observed in NK cells cultured with OCs in comparison to those cultured alone or with DCs (Fig. 4A). Next, we determined the subpopulations of CD16, and CD3 expressing cells within the NK cells cultured alone, or with OCs, or with DCs and counted the numbers of NK and T cells within total lymphocytes. Significantly higher NK cell counts (Fig. 4B) and lower T cell counts (Fig. 4C) were observed in the presence of OCs versus DCs. OC-expanded NK cells displayed significantly higher levels of cytotoxicity against oral squamous cancer stem-like cells (OSCSCs) (Fig. 4D,E). Additionally, NK cells cultured with OCs secreted significantly higher levels of IFN-γ than those cultured with DCs (Fig. 4F).

In addition, we characterized the subpopulations of T cells expanded within the NK cell cultures with OCs or DCs and found that DCs preferentially expanded CD4+ T cells (Fig. 4G,I,J,M) whereas OCs favored the expansion of CD8+ T cells (Fig. 4H-J,M). T cells expanded in NK cell cultures with OCs similar to those expanded by DCs did not express either killer cell lectin-like receptor G1 (KLRG1) or T cell immunoglobulin/mucin domain-containing protein 3 (TIM3), whereas they had similar levels of PD-1 (Fig. 4J). Thus, no significant levels of these check point inhibitors could be seen on T cells expanded by either OC- or DC-expanded NK cells (Fig. 4J). We also noticed slight differences in the expression levels of CD4, CD8, KLRG1, TIM3, and PD-1 in purified T cells cultured with OCs versus DCs in the absence of NK cells (Fig. 4K). T cells in NK + OC co-cultures expressed higher levels of CD45RO; lower levels of CD62L, CD28, CCR7, and CD127; and similar levels of CD44 when compared to NK + DCs co-cultures (Fig. 4L–M). When purified T cells were expanded in the presence of OCs, we observed slightly higher levels of CD45RO and CD28; lower levels of CD62L and CCR7; and similar levels of CD127 and CD44 when compared to those expanded in the presence of DCs (Fig. 4L–M).

Correspondingly, higher levels of cytokines and chemokine secretions were seen in NK cells in comparison to T cells when both were cultured with OCs (Fig. S4 and please check description in supplementary file for Fig. S4). NK cells secreted higher levels MIP-1a, MIP-1B, sCD137, FasL, GMCSF, IFN-γ, sFas, and perforin when compared to T cells (Figs. S4A–S4C, please check description in supplementary file for Fig. S4). CD8+ T cells sorted out from OC-expanded NK cells culture secreted higher levels of GMCSF, sCD137, IFN-γ, FasL, IL-10, and TNF-α when compared to OC-expanded CD8+ T cells in the absence of NK cells (Fig. S4D).

### OCs induce higher cell expansion and IFN-γ secretion in CD8+ T cells than in CD4+ T cells

**Figure 5:**
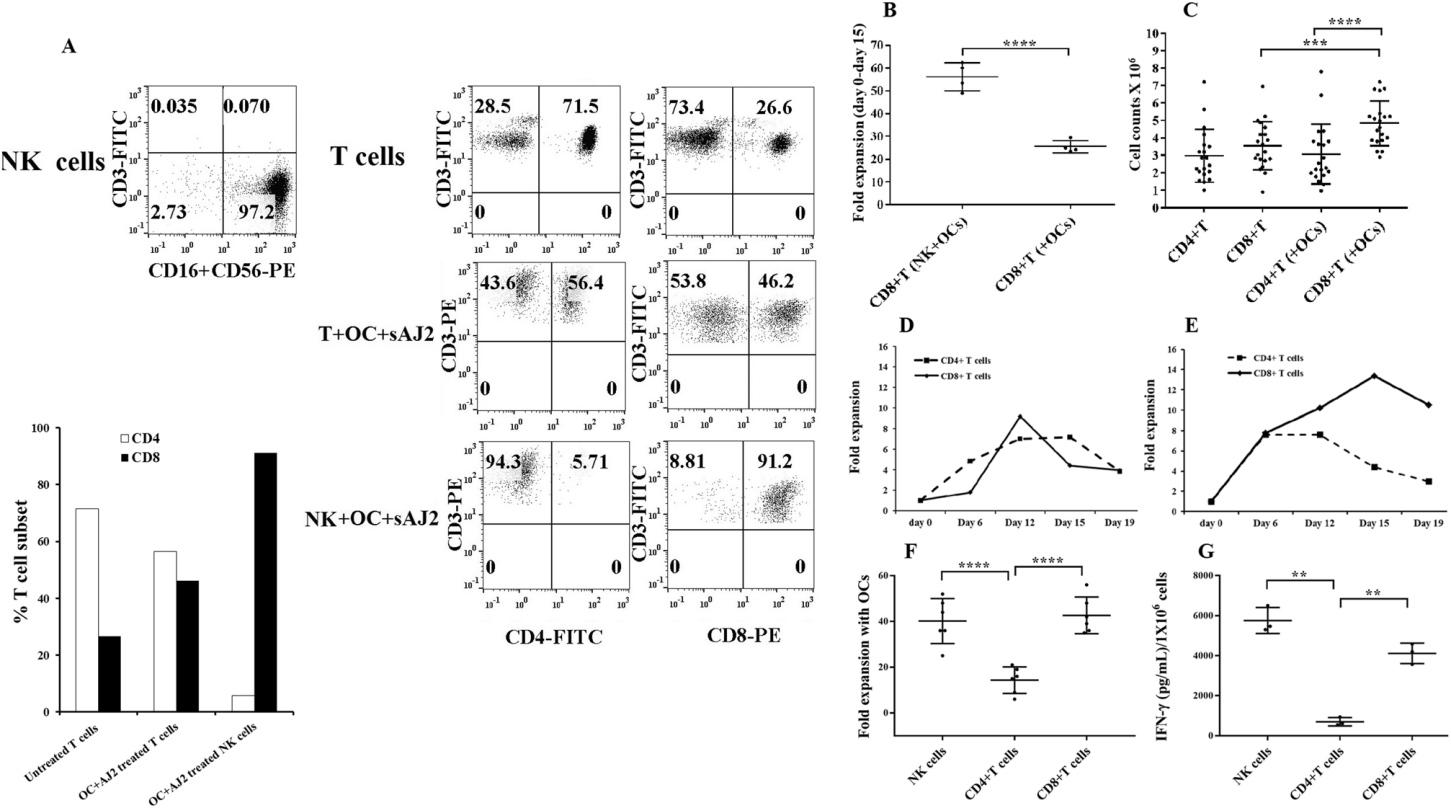
OC-induced activation increases CD8 + T cells. NK cells and T cells were purified from healthy individuals’ PBMCs and the surface expressions of CD3, CD16, and CD56 on freshly isolated NK cells (left panel) and of CD3, CD4 and CD8 on freshly isolated T cells (upper right panel) were determined using flow cytometry. NK cells were treated and co-cultured with OCs and sAJ2 as described in Fig. 1A (middle right panel), and T cells were treated and co-cultured with OCs and sAJ2 as described in Fig. 1E (lower right panel). Surface expressions of CD3, CD4, and CD8 were analyzed on day 12 of the co-culture using flow cytometry **(A)**. NK cells were treated and co-cultured with OCs and sAJ2 as described in Fig. 1A (left bar). Freshly purified CD8 + T cells (1 × 10^6^ cells/ml) from healthy individuals were treated with a combination of IL-2 (100 U/ml) and anti-CD3 (1 µg/ml)/CD28mAb (3 µg/ml) for 18 h before they were co-cultured with OCs and sAJ2 at 1:2:4 ratios (OCs:CD4T or CD8T:sAJ2) (right bar). On days 6, 9, 12 and 15 of the co-cultures, the surface expressions of CD3 + CD8 + T cells were determined using flow cytometry, and the percentages were used to determine the numbers of CD8 + T cells within the total cells. Fold expansion for each time point is shown in the figure (n = 4) **(B)**. Freshly purified CD8 + T cells and CD4 + T cells (1 × 10^6^ cells/ml) from healthy individuals were treated with a combination of IL-2 (100 U/ml) and anti-CD3 (1 µg/ml)/CD28mAb (3 µg/ml) for 18 h before they were co-cultured with sAJ2 (T:sAJ2; 1:2) or with OCs and sAJ2 at 1:2:4 ratios (OCs:CD4T or CD8T:sAJ2). On days 6, 12, 15, and 19 of co-culture, the expanded cells were counted using microscopy (n = 20) **(C)**. Purified CD8 + T cells and CD4 + T cells were treated and co-cultured with sAJ2 as described in Fig. 5C. On days 6, 12, 15 and 19 of co-culture, the expanded cells were counted using microscopy. Fold expansion for each time point is shown in the figure **(D)**. Freshly purified CD8 + T cells and CD4 + T cells were treated and co-cultured with sAJ2 and OCs as described in Fig. 5C. On days 6, 12, 15 and 19 of co-culture, the expanded cells were counted using microscopy. Fold expansion for each time point is shown in the figure **(E)**. NK cells were treated and co-cultured with OCs as described in Fig. 1A**.** CD8 + T cells and CD4 + T cells were treated as described in Fig. 5C. On days 6, 9, 12 and 15 of co-culture, the expanded cells were counted using microscopy. The numbers of OC-expanded NK, OC-expanded CD4 + T and OC-expanded CD8 + T cells were subtracted from the number of non-OC-expanded control cells and the fold expansions were determined by dividing the resulting value by the initial input cells (n = 6) **(F)**. NK cells were treated and co-cultured with OCs as described in Fig. 1A. CD8 + T cells and CD4 + T cells were treated as described in Fig. 5C. The supernatants were harvested from the co-cultures on days 6, 9, 12, and 15 and the secretions of IFN-γ were determined using single ELISA, and the values were adjusted based on a million lymphocytes (n = 3) **(G)**.

OCs were found to induce higher expansion of CD8+ T cells in NK cultures when compared to those with purified T cells (Fig. 5A and S5) or purified CD8+ T cells (Fig. 5B). No significant differences in the degree of expansion could be seen when purified CD4 + and CD8+ T cells were treated with anti-CD3/CD28 antibody + IL-2 and cultured in the absence of OCs (Fig. 5C,D). However, in contrast to CD4+ T cells, a continuous rise in the fold expansion of CD8+ T cells could be seen when the cells were treated with anti-CD3/CD28 antibody + IL-2 and cultured with OCs (Fig. 5C,E). Under the same experimental condition CD4+ T cell counts increase initially, but plateaued soon after, and then declined after day 12 of culture (Fig. 5E). We then compared the expansion and secretion of IFN-γ by the NK, CD4+ T, and CD8  T cells after they were treated as described above and cultured with OCs. Higher cell expansion (Fig. 5F) and IFN-γ secretion (Fig. 5G) could be observed in NK and CD8+ T cells as compared to CD4+ T cells.

### Increased CD8+ T cells, IFN-γ secretion, and cytotoxicity in various tissue compartments of oral tumor-bearing hu-BLT mice in response to NK cell immunotherapy

**Figure 6:**
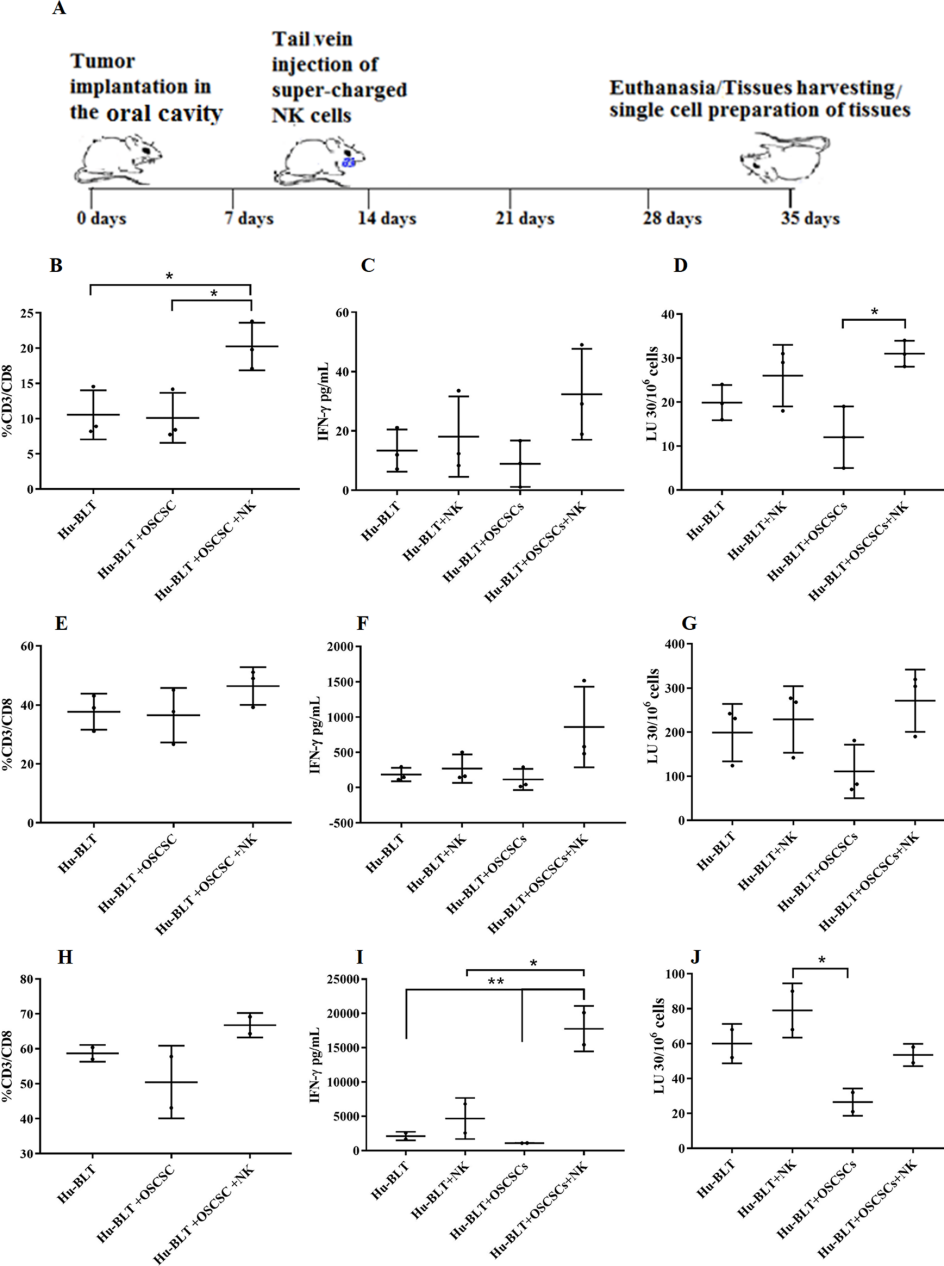
OC-expanded NK cell immunotherapy increased CD8 + T cells, IFN-γ secretion, and NK cell-mediated cytotoxicity in BM, spleen, and peripheral blood of hu-BLT mice. Hu-BLT mice were orthotopically injected with 1 × 10^6^ human OSCSCs into the floor of the mouth. One to two weeks after the tumor implantation, mice received OC-expanded NK cells via tail-vein injection. The disease progression and weight loss were monitored for another 3–4 weeks (n = 3) **(A)**. Hu-BLT mice were implanted with OSCSC tumors and were injected with NK cells as depicted in Fig. 6A. At the end of experiment, hu-BLT mice were sacrificed; the spleens, BM, and peripheral blood were harvested; and single cell suspensions were obtained and cultured as described in the Materials and Methods section. Surface expressions of CD3 and CD8 were analyzed on day 7 of BM cultures (n = 3) **(B)**, spleen cultures (n = 3) **(E)**, and PBMC cultures (n = 2) **(H)** using flow cytometry. The supernatants were harvested from the cultures on day 7 of BM culture (n = 3) **(C)**, spleen culture (n = 3) **(F)**, and PBMC culture (n = 2) **(I),** and the secretions of IFN-γ were determined using single ELISA. Cytotoxicity of day 7 cultured BMs (n = 3) **(D)**, spleens (n = 3) **(G)**, and PBMCs (n = 2) **(J)** were determined against OSCSCs using standard 4-h ^51^Cr release assay. LU 30/10^6^ cells were determined using the method described in Fig. 1B.

Hu-BLT mice were implanted with OSCSCs in the oral cavity and injected with OC-expanded NK cells with potent cytotoxic and cytokine secretion capabilities. After 4–5 weeks, the mice were sacrificed and tissues were harvested and dissociated in order to obtain single-cell suspensions for analysis (Fig. 6A). We observed increased proportions of CD3+ CD8+ T cells in the BM (Fig. 6B), spleen (Fig. 6E), and peripheral blood (Fig. 6H) of tumor-bearing mice injected with OC-expanded NK cells when compared to tumor-bearing mice injected with vehicle only or healthy non-tumor bearing mice. NK cell immunotherapy also augmented the IFN-γ secretion and NK cell-mediated cytotoxicity in BM (Fig. 6C,D), spleen (Fig. 6F,G), and peripheral blood (Fig. 6I,J) in tumor-bearing mice. Increased secretion of IFN-γ, IL-6, and ITAC and decreased secretion of IL-8 and GM-CSF were also seen in sera harvested from the peripheral blood of tumor-bearing mice injected with OC-expanded NK cells versus those injected with vehicle alone or non-tumor bearing mice injected with OC-expanded NK cells (Fig. S6).

### NK cells preferentially lyse CD4+ T cells when compared to CD8+ T cells

**Figure 7:**
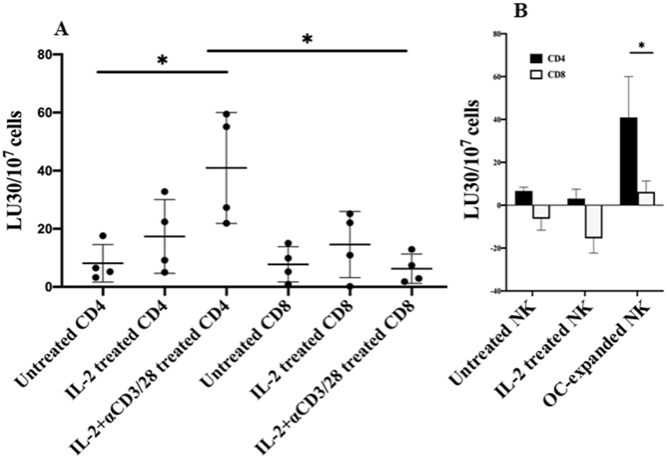
NK cells preferentially lyse CD4 + T cells and not CD8 + T cells. Freshly purified CD4 + T and CD8 + T cells from healthy individuals were left untreated, treated with IL-2 (100 U/ml), or treated with a combination of IL-2 (100 U/ml) and anti-CD3 (1 µg/ml)/CD28mAb (3 µg/ml) for 18 h. NK cells which were treated and co-cultured with OCs as described in Fig. 1A, were used to determine cytotoxicity against CD4 + and CD8 + T cells using a TVA dye assay. LU 30/10^7^ cells were determined using the inverse of the number of NK cells required to lyse 30% of target cells × 1000 (n = 4) **(A)**. NK cells were left untreated or treated with IL-2 (1000 U/ml) for 18 h, or expanded with OCs as described in Fig. 1A, and used to determine cytotoxicity against IL-2 (100 U/ml) and anti-CD3 (1 µg/ml)/CD28mAb (3 µg/ml) Treated CD4 + and CD8 + T cells using a TVA dye assay. LU 30/10^7^ cells were determined as described in Fig. 7A. One of several representative experiments is shown in the figures **(B)**.

NK cell-mediated cytotoxicity against CD4+ and CD8+ T cells were assessed using TVA dye. OC-expanded NK cells preferentially lysed CD4+ T cells but not CD8+ T cells (Fig. 7A) and the levels were higher than those mediated by the IL-2 treated primary NK cells (Fig. 7B). Similarly, IL-2+ anti-CD16mAb treated NK cells were able to lyse CD4+ T cells when compared to CD8+ T cells (Fig. S7).

## Discussion

The dynamics of NK cell mediated regulation and activation of CD4+ and CD8+ T cells have not been explored fully and are the subjects of this paper. We have previously shown that NK functional inactivation and loss of numbers occurs at both the pre-neoplastic and neoplastic stages of pancreatic cancer due to the effects of both the KRAS mutation and high fat calorie diet^55–57^. In this report we also demonstrate that patients with pancreatic cancer as well as a few other cancers have severely suppressed NK function. Both cytotoxicity and the ability to secrete IFN-γ are suppressed in cancer patients’ NK cells. In addition, we also demonstrate that the percentages of NK, monocyte, and CD11b+ immune cells are increased in cancer patients, even though the total numbers of PBMCs are severely decreased. In addition, the percentages of CD3+ T cells and B cells are substantially decreased. Thus, although the percentages of NK cells are elevated in cancer patients, the function of NK cells are severely depressed, indicating a profound immunosuppression of NK cells from cancer patients. Even when NK cells were purified and expanded and super-charged by the use of OCs^15,56,57^, the cells from cancer patients had much lower ability to expand and mediate cytotoxicity and secrete IFN-γ when compared to those expanded from healthy individuals. Thus, lower recovery of PBMCs from cancer patients could partly be due to the inability of different lymphocyte subsets such as NK cells to proliferate and expand when compared to those expanded from healthy individuals. Both primary and OC-expanded NK cells from cancer patients are defective in their function, therefore, the defects observed in patients’ primary NK cells are dominant and are only moderately improved when these cells are expanded in the presence of allogeneic OCs^15^. Expansion of patients’ T cells as well as IFN-γ secretion from OC-expanded T cells are also decreased under different activation conditions (Fig. 1E–G, S3A-S3C, and S3E). Cancer patients’ immune effectors demonstrated higher percentages of CD45RO and decreased percentages of CD62L surface expressions indicating the increased status of immune activation in vivo. This is also evident from the increased percentage of CD8+ T cells and decreased ratios of CD4+/CD8+ T cells in patients (Fig. 3B,C).

Increased percentages of NK cells in cancer patients can be one reason why we see preferential increase in CD8+ T cells and lower ratios of CD4+/CD8+ T cells. Our studies indicate that NK cells are very important in the preferential expansion of CD8+ T cells. In particular, OCs are important in the expansion of NK cells as reported previously^15,56,57^. The majority of T cells expanded by the NK cells are CD8+ T cells, and similar profile of CD8+ T cell expansion by the NK cells is seen when NK cells are obtained from both healthy individuals and cancer patients indicating that NK cells are indispensable for the expansion of CD8+ T cells. Although OCs have some effect on the decreased ratios of CD4+ to CD8+ T cells in both healthy individuals and cancer patients T cells, the ratios are substantially decreased in the presence of NK cells indicating higher selection and expansion of CD8+ T cells and loss of CD4+ T cells by the expanded NK cells (Fig. 3E).

The patients are found to have on average higher percentages of CD8+ T cells in their peripheral blood when compared to those obtained from healthy individuals (Fig. 3B,C). When OC-derived from healthy individuals were used to expand both healthy individuals and cancer patients’ T cells, OC-expanded T cells also demonstrated higher percentages of CD8+ T cell expansion from both healthy and patient derived T cells, which were higher than those seen from those obtained initially from the peripheral blood (Fig. 3B–E). Thus, the higher expansion seen with cancer patients’ OC-expanded T cells is likely due to the higher frequencies of CD8+ T cells in the patients when compared to healthy individuals. Indeed, on average 1 percent primary CD8+ T cells when expanded by healthy OCs will give rise to 1.2 percent expanded CD8+ T cells by cancer patients’ T cells, but that average is at 1.6 percent with T cells from healthy individuals, which is higher. Thus, anti-CD3/CD28 antibody activation and signaling through T cells augments the percentages of CD8+ T cells moderately, and the levels of expansion are less by cancer patients’ CD8+ T cells when compared to CD8+ T cells expanded from healthy individuals (Fig. 3D,E). In addition, OC-expanded NK cells expanded 2.73 percent CD8+ T cells from 1 percent of CD8+ T cells from healthy individuals, whereas from cancer patients those percentages remained lower at 1.75 percent which on average is an almost one percentage point difference (Fig. 3B–E). Thus, the extent of CD8+ T cell selection and expansion by OC-expanded NK cells is much higher in healthy individuals than those obtained from cancer patients. Based on these results, it is evident that the selection and expansion of CD8+ T cells from both OC-expanded T and OC-expanded NK cells is significantly inferior by the cancer patients cells when compared to those of healthy individuals.

We also determined the rate of OC mediated CD8+ T cell expansion from both healthy individuals and cancer patients using autologous OCs. Cancer patients’ OCs had lower ability to expand autologous CD8+ T cells from OC-expanded T and NK cells and the percentages of expansion were much less when compared to CD8+ T cells expansion from OC expanded T and NK cells from healthy individuals in an autologous system (Fig. S5). In addition, when the numbers of expanded NK cells and the levels of cytotoxicity and secretion of IFN-γ were assessed in an autologous system much lower levels of expansion, cytotoxicity and IFN-γ secretion could be observed from patient OC-expanded NK cells when compared to OC-expanded NK cells from healthy individuals (Fig. 2E–G). As expected, using cancer patients’ OCs for the expansion of NK cells from healthy individuals or healthy OCs with cancer patients’ NK cells, we observed much lower expansion and function when compared to those obtained from OC-expanded NK cells in healthy individuals in an autologous system (Fig. 2). The lowest levels of expansion and function compared to the three groups mentioned above were seen when cancer patients’ OCs were used to expand autologous NK cells, indicating that there are functional deficiencies in both NK cells and OCs from cancer patients, when compared to the function of both cell types from healthy individuals. Thus, these factors should be considered when designing immunotherapeutic strategies using autologous and allogeneic NK cells since even when one provides the best expansion and the highest quality of allogeneic NK cells to cancer patients, the expansion and function of those NK cells will be short lived since the supporting cells are also defective and will likely not provide the adequate signals for the continued expansion of NK cells. Indeed, when comparing the surface receptor expression between patient and healthy OCs, there is significant down-modulation of activating receptors which could be one reason why the patient OCs may not support the expansion of allogeneic or autologous NK and CD8+ T cells^56^. However, we also see a substantial decrease in MHC-class I inhibitory signals which should provide an activating signal due to decrease binding and inhibition of NK cells through MHC-class I inhibitory receptors. It appears that most receptors are down-modulated on the surface of cancer patients’ OCs irrespective of whether they are activating or inhibitory ligands for the NK cells^56^. In this scenario it is possible that lack of activating ligands supersedes the effect of lack of inhibitory ligands since OCs from cancer patients are not able to activate autologous NK cells.

In agreement with our in vitro studies, injection of OC-expanded NK cells to tumor-bearing hu-BLT mice increased the numbers of CD8+ T cells in BM, spleen, and peripheral blood resulting in the increased levels of NK cell-mediated cytotoxicity as well as increased secretion of IFN-γ (Fig. 6B–J). Increased levels of IFN-γ, IL6, ITAC were also observed in the sera of tumor-bearing hu-BLT mice injected with OC-expanded NK cells (Fig. S6).

Significant differences are observed between DC-induced expansion of NK cells and OC-induced NK cell expansion. OC-induced expansion of NK cells increased CD8+ T cell expansion, whereas DC-induced expansion of NK cells resulted in expansion of CD4+ T cells. At the moment, the mechanisms governing the differential expansion of CD4+ vs. CD8+ T cells by DC- vs. OC-expanded NK cells respectively are not fully understood. However, there is larger increases in percentages of CD45RO and a higher decrease in percentages of CD62L surface expressions in T cells expanded by OC-expanded NK cells than DC-expanded NK cells indicating higher activation of T cells by the OC-expanded NK cells when compared to those induced by DC-expanded NK cells (Fig. 4L).

It is possible that the higher activation signals by the OC-expanded NK cells are necessary for greater expansion of CD8+ T cells than CD4+ T cells. Indeed, OC-induced expansion of CD8+ T cells when total CD3+ T cells were used for expansion resulted in moderate increase in the expansion of CD8+ T cells and in the slight decline of CD4+ T cells (Figs. 3D,5A). Therefore, signals from both OCs and NK cells appear to be important in increased expansion and activation of CD8+ T cells, although the effect of NK cells appears to be more dominant than OCs. Indeed, NK cells expanded by OCs have greater cytotoxic activity than those expanded by the DCs, potentially providing the underlying mechanism for targeting of CD4+ T cells and sparing of CD8+ T cells. In support of this mechanism of action we have also observed that OC-expanded NK cells differentially targeted activated CD4+ and CD8+ T cells (Figs. 7 and S7). The OC-expanded NK cells as well as IL-2 + anti-CD16mAb treated NK cells but less primary IL-2 activated NK cells were able to target activated CD4+ T cells (Figs. 7 and S7). Indeed, it has previously been shown that NK cells inhibit proliferation of CD4+ T cells under chronic antigen stimulation in the model of GVHD through Fas receptor and not perforin mediated killing, and that the lysis was mediated through the NKG2D ligand expression^43^. In agreement, CD56^bright^ NK cells in comparison to CD56^dim^ subset were found to have higher degranulation and lysis of activated CD4+ T cells^42^. CD56^bright^ NK cells were previously shown by many laboratories to have higher secretion of cytokines in the presence of no or lower cytotoxicity similar to those found with IL-2+ anti-CD16mAb treated NK cells which we have previously coined as split anergized NK cells^58,59^. Therefore, it is possible that the underlying mechanisms of CD4+ T cell lysis is through their death receptors triggered by Fas ligand, TNF-a and TRAIL on NK cells^60^. Indeed, both split anergized NK cells^59^ and OC-expanded NK cells have very high induction of Fas ligand and TNF-α (Fig. S4). However, since super-charged NK cells have also significantly higher granule content with potent cytotoxic function (manuscript in prep), the granule mediated lysis of CD4+ T cells can’t be ruled out in our system at present. Furthermore, it was also shown that upon stimulation with antigen and co-stimulatory signals CD4+ T cells undergo activation induced cell death through Fas receptors whereas CD8+ T cells are rendered non-responsive but gain function when IL-2 is provided^60^. Therefore, there are clear differences between CD4+ and CD8+ T cells subsets in their susceptibility to cell death and mode of expansion. Thus, greater expansion of CD8+ T cells by both OCs and NK cells suggests increased selection as well as expansion of CD8+ T cells since NK cells select and also trigger expansion of CD8+ T cells. On the other hand, it appears that OCs will only aid moderately in selection and expansion since these cells were not shown to have cytotoxic capability. Whether there are differences between the CD4+ and CD8+ T cells in the magnitude of expansion also requires further studies. It is also possible that important cytokines such as IFN-γ and TNF-α secreted by either split-anergized NK cells or OC-expanded super-charged NK cells are able to expand the CD8+ T cells after their selection (Fig. S4, and ref^61,62^.).

**Figure 8:**
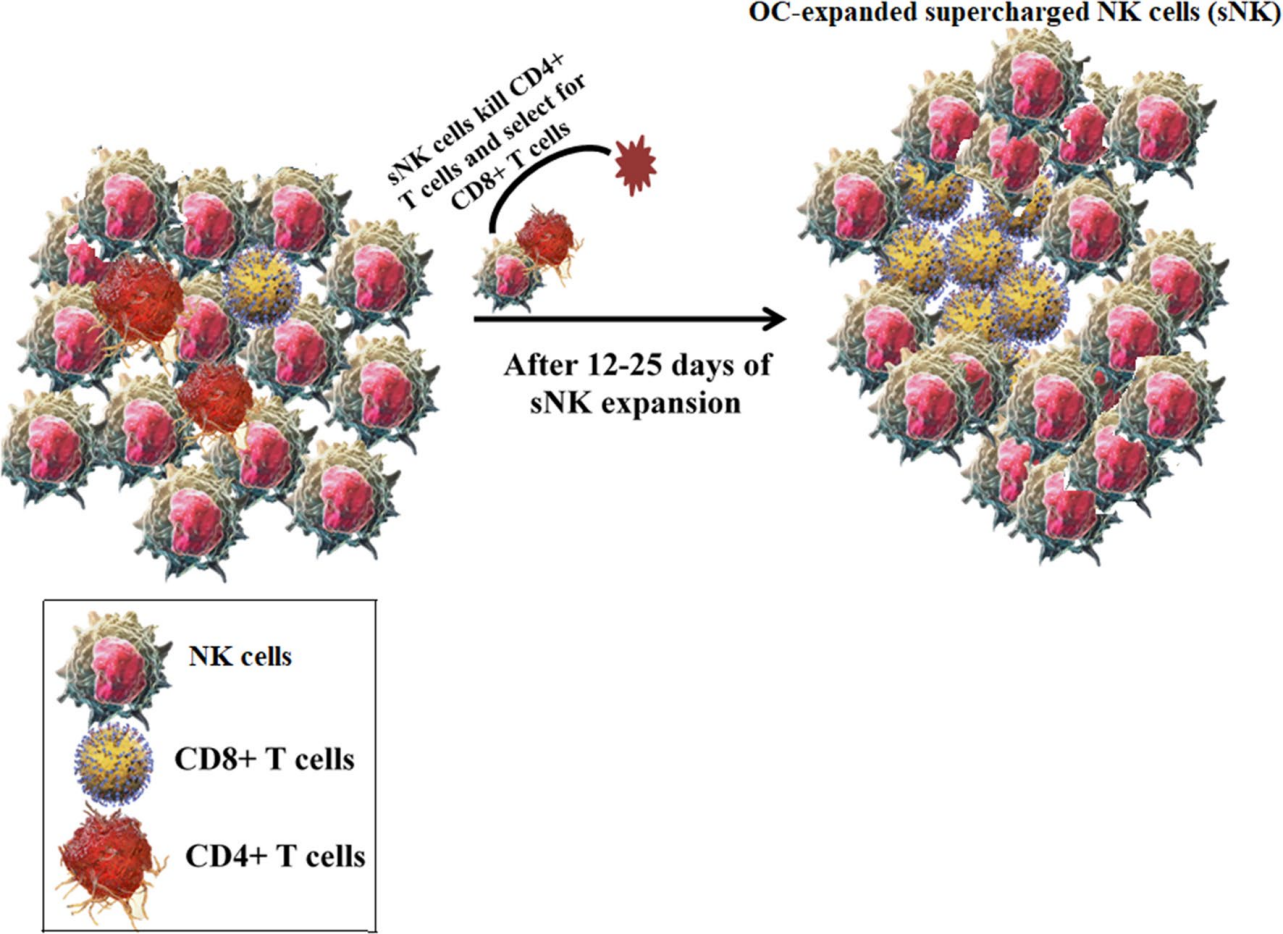
Schematic representation of mechanisms for the selection and expansion of CD8 + T cells by OC-expanded supercharged NK cells. Freshly purified NK cells with 1–3% contaminated T cells (CD4 + and CD8 + T cells) were treated and co-cultured with OCs as described in Materials and Methods (left panel). After days 12–25 of co-culture, the OC-expanded cells express approx. 20% T cells majority of which are CD8 + T cells (right panel). The expansion of CD8 + T cells is likely due to the lysis of CD4 + T cells by the super-charged NK cells.

Although OCs were able to expand CD8+ T cells somewhat, the expansion of these cells were significantly accelerated in the presence of OC-expanded NK cells (Figs. 3D,E and 5A,B). Therefore, there could be potentially two different mechanisms of CD8+ T cells expansion by the OC-expanded NK cells. One mechanism is likely contributed by the OCs in the initial phases of expansion where there still remains some OCs in the NK cultures which could be approximately up until day 6 or maximum 9 of expansion with fewer or minor expansion of CD8+ T cells. By day 9 no OCs are remaining in the culture of NK cells and therefore, there are only expanding super-charged NK cells with more rapidly expanding CD8+ T cells. Thus, the second mechanism is contributed by the super-charged NK cells which are likely through targeting of remaining CD4+ T cells and selection of CD8+ T cells and activation of CD8+ T cells. Although there are significant numbers of CD4+ T cells remaining after OC-mediated expansion of T cells, in the presence of OC-expanded NK cells the majority if not all are primarily CD8+ T cells (Fig. 3D). At the moment it is not clear how OCs contribute to the preferential expansion of CD8+ T cells. As for OC-expanded NK mediated expansion of CD8+ T cells, a significantly higher activation of CD8+ T cells in terms of increased percentage of cells expressing CD45RO and lower percentage of cells expressing CD62L is seen with OC-expanded NK cells when compared to just OC activated T cells (Fig. 4L,M), therefore, it is possible that either NK cells differentially target and kill activated CD4+ T cells and/or that activation induced cell death is higher in CD4 + T cells than it is in CD8 + T cells (Fig. 8). The higher activation of CD8+ T cells by the NK cells in comparison to OC-induced CD8+ T cells is also seen when different cytokine levels were assessed (Fig. S4D).

Potential relevance of our observations could be seen in the studies reported with multiple myeloma (MM) patients. Indeed, these patients have multifocal neoplastic proliferation of monoclonal plasma cells in the bone marrow where significant numbers of OCs reside. It was shown that these patients had higher levels of NK and CD8+ T cells in both peripheral blood and bone marrow aspirates when compared to heathy controls^12^. It was also found that the ratio of CD4+/CD8+ T cells was decreased in the patients and this decrease was co-related with an increase in human leukocyte antigen (HLA)-DR expression by CD8+ but not CD4+ T cells^63^. Moreover, it was noted that patients with long-term disease control exhibited an expansion of cytotoxic CD8+ T cells and NK cells^9^. T cell expansions in MM patients have a phenotype of cytotoxic T cells, with expanded V-beta TCR populations having predominantly CD8+ , CD57+, CD28- and perforin + phenotype^11^. Although our observations are likely relevant to MM patients since they exhibit significant BM pathology, it is also likely that the mechanisms discussed in this paper can also occur in patients who may sustain bone metastasis or have primary tumors inflicting bone. Prostate, breast, and lung cancers have predilection to metastasize to bone^64–67^, although pancreatic and colon cancers can also metastasize to bone^68–71^. Whether these mechanisms are operational in osteoporosis or during non-metastatic bone pathologies should await future studies.

## Materials and methods

### Cell lines, reagents, and antibodies

Oral squamou carcinoma stem cells (OSCSCs) were isolated from patients with tongue tumors at UCLA^59,72–74^. OSCSCs were cultured in RPMI 1640 (Life Technologies, CA, USA) supplemented with 10% fetal bovine serum (FBS) (Gemini Bio-Product, CA, USA). RPMI 1640 supplemented with 10% FBS was used to culture human NK cells, human T cells, and hu-BLT mice BM, spleen and PBMCs. Alpha-MEM (Life Technologies, CA, USA) supplemented with 10% FBS was used for osteoclast (OCs) and dendritic cell (DCs) cultures. M-CSF, anti-CD16 mAb, and flow cytometric antibodies were purchased from Biolegend, CA, USA. RANKL, GM-CSF, and IL-4 were purchased from PeproTech, NJ, USA, and recombinant human IL-2 was obtained from Hoffman La Roche, NJ, USA. Human anti-CD3/CD28 was purchased from Stem Cell Technologies, Vancouver, Canada. Probiotic bacteria, AJ2 is a combination of eight different strains of gram-positive probiotic bacteria (*Streptococcus thermophiles, Bifidobacterium longum, Bifidobacterium breve, Bifidobacterium infantis, Lactobacillus acidophilus, Lactobacillus plantarum, Lactobacillus casei, and Lactobacillus bulgaricus*) elected for their superior ability to induce optimal secretion of both pro-inflammatory and anti-inflammatory cytokines in NK cells^75^. RPMI 1640 supplemented with 10% FBS was used to re-suspend AJ2. Human ELISA kits for IFN-γ were purchased from Biolegend, CA, USA. Phosphate buffered saline (PBS) and bovine serum albumin (BSA) were purchased from Life Technologies, CA, USA. Matrigel was purchased from Corning, NY, USA.

### Purification of human NK cells, T cells and monocytes

Written informed consents were obtained from healthy donors and cancer patients as approved by the UCLA Institutional Review Board (IRB) (Table S1), and all procedure were approved by UCLA-IRB and all methods were carried in accordance with UCLA-IRB guidelines and regulations. Peripheral blood mononuclear cells (PBMCs) were isolated from peripheral blood as described before^76^. Briefly, PBMCs were obtained after Ficoll-hypaque centrifugation and were used to isolate NK cells, T cells, CD4+ T cells, CD8+ T cells, and monocytes using the EasySep Human NK cell, EasySep Human T cell, EasySep Human CD4 T, and EasySep Human CD8 T cell, EasySep Human monocytes enrichments kits, respectively, purchased from Stem Cell Technologies, Vancouver, BC, Canada. Isolated NK cells, T cells, CD4+ T cells, CD8+ T cells, and monocytes were stained with anti-CD16, anti-CD3, anti-CD4, anti-CD8, anti-CD14 antibodies, respectively, to measure the cell purity using flow cytometric analysis.

### Generation of human OCs and DCs

To generate OCs, monocytes were cultured in alpha-MEM media supplemented with M-CSF (25 ng/mL) and RANKL (25 ng/mL) for 21 days, media was replenished every three days. OCs were tested using TRAP staining to confirm multinucleated cells as described previously^13^. Monocytes were cultured in alpha-MEM media supplemented with GM-CSF (150 ng/mL) and IL-4 (50 ng/mL) for 7 days to generate DCs.

### Sonication of probiotic bacteria (AJ2)

AJ2 bacteria were weighed and re-suspended in RPMI 1640 medium containing 10% FBS at a concentration of 10 mg/ml. The bacteria were thoroughly vortexed, then sonicated on ice for 15 s at 6 to 8 amplitudes, sonicated samples were then incubated for 30 s on ice, cycle was repeated for five rounds. After every five rounds of sonication, we checked each sample under the microscope until at least 80% of bacterial walls were lysed. It was determined that approximately 20 rounds of sonication/incubation on ice were necessary to achieve complete sonication. Finally, the sonicated AJ2 (sAJ2) were aliquoted and stored at -80˚C until use.

### Expansion of human NK cells and human T cells

Human purified NK cells were activated with rh-IL-2 (1000 U/ml) and anti-CD16 mAb (3 µg/ml) for 18–20 h before they were co-cultured with feeder cells (OCs or DCs) and sAJ2 (OCs:NK:sAJ2 or DCs:NK:sAJ2; 1:2:4) in RPMI 1640 medium containing 10% FBS. The medium was refreshed every three days with RPMI containing rh-IL-2 (1500 U/ml). Purified human T cells were activated with rh-IL-2 (100 U/ml) and anti-CD3 (1 µg/ml)/anti-CD28 antibody (3 µg/ml) for 18–20 h before they were co-cultured with OCs or DCs and sAJ2 (OCs:T:sAJ2 or DCs:T:sAJ2; 1:2:4) in RPMI 1640 medium containing 10% FBS. The culture media was refreshed with rh-IL-2 (150 U/ml) every three days.

### Enzyme-Linked Immunosorbent Assays (ELISAs) and multiplex cytokine assay

Single ELISAs and multiplex assays were performed as previously described^76^. To analyze and obtain the cytokine and chemokine concentration, a standard curve was generated by either two- or three-fold dilution of recombinant cytokines provided by the manufacturer. For multiple cytokine array, the levels of cytokines and chemokines were examined by multiplex assay, which was conducted as described in the manufacturer’s protocol for each specified kit. Analysis was performed using a Luminex multiplex instrument (MAGPIX, Millipore, Billerica, MA), and data was analyzed using the proprietary software (xPONENT 4.2, Millipore, Billerica, MA).

### ^51^Cr release cytotoxicity assay

The ^51^Cr release assay was performed as described previously^77^. Briefly, different numbers of effector cells were incubated with ^51^Cr-labeled target cells. After a 4-h incubation period, the supernatants were harvested from each sample and the released radioactivity was counted using the gamma counter. The percentage specific cytotoxicity was calculated as follows:

$$\%{\text{Cytotoxicity}}={\frac{{\text{Experimental cpm}}-{\text{spontaneous cpm}}} {{\text{Total cpm}}-{\text{spontaneous cpm}}}}$$

LU 30/10^6^ is calculated by using the inverse of the number of effector cells needed to lyse 30% of tumor target cells × 100.

### Surface staining assay

For surface staining, the cells were washed twice using ice-cold PBS + 1%BSA. Predetermined optimal concentrations of specific human monoclonal antibodies were added to 1 × 10^4^ cells in 50 µl of cold PBS + 1%BSA, and were incubated on ice for 30 min. Thereafter cells were washed in cold PBS + 1%BSA and brought to 500 µl with PBS + 1%BSA^57^. Flow cytometric analysis was performed using Beckman Coulter Epics XL cytometer (Brea, CA), and results were analyzed in the FlowJo vX software (Ashland, OR)^57^.

### Tumor implantation in hu-BLT mice

Animal research was performed under the written approval of the UCLA Animal Research Committee (ARC) in accordance with all federal, state, and local guidelines. Combined immunodeficient NOD.CB17-Prkdcscid/J and NOD.Cg-Prkdcscid Il2rgtm1Wjl/SzJ (NSG lacking T, B, and NK cells) were purchased from Jackson Laboratory. Humanized-BLT (hu-BLT; human bone marrow/liver/thymus) mice were prepared on NSG background as previously described^78,79^. To establish orthotopic tumors, mice were first anesthetized with isoflurane in combination with oxygen, and 1 × 10^6^ human OSCSC tumor cells suspended in 10 μl HC Matrigel were then injected directly into the floor of their mouths. One to two weeks after tumor implantation mice received 1.5 × 10^6^ OC-expanded NK cells via tail vein injection (Fig. 6A). Four to five later, mice were euthanized when signs of morbidity were evident and bone marrow, spleen, and peripheral blood were harvested.

### Cell isolation and cell cultures of hu-BLT mice’ BM, spleen, and peripheral blood

To obtain single-cell suspensions from BM, femurs were cut at both ends and flushed through using RPMI 1640 media; afterwards, BM cells were filtered through a 40 µm cell strainer. To obtain single-cell suspensions from spleen, the spleens were minced, and the samples were filtered through a 40 µm cell strainer and centrifuged at 1500 rpm for 5 min at 4˚C. The pellet was re-suspended in ACK buffer for 2–5 min to remove the red blood cells followed by re-suspension in RPMI media and centrifugation at 1500 rpm for 5 min at 4˚C. PBMCs were isolated from peripheral blood using Ficoll-Hypaque centrifugation of heparinized blood specimens. The buffy coats containing PBMCs were harvested, washed, and re-suspended in RPMI 1640 medium. Cells obtained from each tissue sample were treated with IL-2 (1000 U/ml) and cultured in RPMI 1640 medium containing 10% FBS for 7 days.

### Target cell visualization assay (TVA)

Target cells were incubated with TVA dye at 37 ˚C for 15 min and then cultured with effector cells for 4 h. Afterwards, the target cells were counted with ImmunoSpot S6 universal analyzer/software (Cellular Technology Limited, OH, USA) at 525 nm emission wavelengths. The percentage specific cytotoxicity was calculated as follows:

$$\% {\text{Cytotoxicity}} = {\frac{{{\text{Experimental cpm}} - {\text{spontaneous cpm}}}} {{{\text{Total cpm}} - {\text{spontaneous cpm}}}}}$$

LU 30/10^7^ is calculated by using the inverse of the number of effector cells needed to lyse 30% of tumor target cells × 100.

### Statistical analyses

All statistical analyses were performed using the GraphPad Prism-8 software. An unpaired or paired, two-tailed student’s t-test was performed for the statistical analysis for experiments with two groups. One-way ANOVA with a Bonferroni post-test was used to compare different groups for experiments with more than two groups. (n) denotes the number of human donors or mice for each experimental condition. Duplicate or triplicate samples were used in the in vitro studies for assessment. The following symbols represent the levels of statistical significance within each analysis: ***(p value < 0.001), **(p value 0.001–0.01), *(p value 0.01–0.05).

## Supplementary information

Supplementary information.
