# Supplementary material for: Osteoclast-expanded super-charged NK-cells preferentially select and expand CD8+ T cells
Source: Sci Rep. 2020 Nov 23;10:20363. doi: 10.1038/s41598-020-76702-1 (PMC7683603; doi:10.1038/s41598-020-76702-1)

**Supplementary data**

**Osteoclast-expanded super-charged NK-cells preferentially select and expand CD8+T cells**

Kawaljit Kaur^1,2^, Meng-Wei Ko^1,2^, Nick Ohanian^1,2^, Jessica Cook^1,2^, and Anahid Jewett^1,2,3*^

^1^Division of Oral Biology and Oral Medicine, School of Dentistry and Medicine, Los Angeles, CA, USA, ^2^The Jane and Jerry Weintraub Center for Reconstructive Biotechnology; UCLA School of Dentistry and Medicine, ^3^The Jonsson Comprehensive Cancer Center, UCLA, Los Angeles, CA, USA.

**Corresponding author:** Anahid Jewett, UCLA School of Dentistry, 10833 Le Conte Ave., Los Angeles, CA 90095, USA; email: [ajewett@ucla.edu](mailto:ajewett@ucla.edu)

**Tel:** 1-310-206-3970, **Fax:** 1-310-794-7109

**Results**

**OC-expanded NK cells secreted more cytokines and chemokines when compared to expanded T cells**

NK cells were cultured with OCs for 12 days before expanded NK cells, and NK cell expanded CD8+ T cells were isolated from the same culture. Isolated NK cells were treated with a combination of IL-2 and anti-CD16 mAb, and NK expanded CD8+ T cells were treated with IL-2 and anti-CD3/CD28 mAb for 18 hours before the supernatants were harvested from the cultures and secretions were assessed using multiplex arrays. We compared the amounts secreted by the NK cells with the amounts which were secreted by the NK-expanded CD8+ T cells, and determined the fold increase in NK cells when compared to NK expanded CD8+ T cells. NK cells secreted higher levels of all cytokines and chemokines with the exception of IL-3 which was lower by the NK cells than NK-expanded CD8+ T cells as shown in the Figure S4A. In particular NK cells secreted higher levels of secretedCD137, secreted Fas-Ligand (sFasL), Granzyme A and B, IL-10, TNF-a, MIP1-1a, and MIP1b, when compared to CD8+ T cells (Fig. S4A). OC-expanded NK cells produced more abundant amounts of GM-CSF, soluble CD137, IFN-γ, soluble Fas, sFasL, perforin, MIP-1a, and MIP1b, while OC-expanded T cells readily produced IL-10, granzymes A and B, and TNF-α during the expansion periods (Figs. S4B-S4C). Then, we examined the secreted factors from CD8+ T cells isolated from day 12 OC-expanded NK cell cultures and compared to purified CD8+ T cells expanded by OC only. CD8+ T cells isolated from the OC-expanded NK cell cultures secreted higher levels of GM-CSF, soluble CD137, IFN-γ, IL-10, sFasL, and TNF-α but lower levels of granzyme A and perforin; and similar levels of granzyme B and soluble Fas, when compared to OC expanded CD8+ T cells (Fig. S4D).

**Material and Methods**

**Sera collection from human donors and hu-BLT mice peripheral blood**

Peripheral blood (200 µl) was collected in 1.5 ml heparin-free Eppendorf tubes and left in room temperature for 15-20 minutes. The tubes were then centrifuged at 2000 rpm for 10 mins, and the sera were then harvested.

**Participating cancer patients**

**Table S1**

|  | **Gender** | **Cancer type** | **Cancer stage (TNM)** |
| --- | --- | --- | --- |
| Patient 1 | M | Oral | Stage 4 |
| Patient 2 | M | Oral | Stage 4 |
| Patient 3 | M | Oral | Stage 4 |
| Patient 4 | F | Pancreatic | Stage 4 |
| Patient 5 | M | Pancreatic | Stage 4 |
| Patient 6 | F | Pancreatic | Stage 4 |
| Patient 7 | F | Pancreatic | Stage 4 |
| Patient 8 | F | Pancreatic | Stage 4 |
| Patient 9 | M | Pancreatic | Stage 4 |
| Patient 10 | M | Pancreatic | Stage 4 |
| Patient 11 | M | Pancreatic | Stage 4 |
| Patient 12 | M | Pancreatic | Stage 4 |
| Patient 13 | M | Colon | Stage 4 |
| Patient 14 | M | Colon | Stage 4 |
| Patient 15 | M | Colon | Stage 4 |
| Patient 16 | M | Prostate | Stage 4 |

**Figure legends**

**Figure S1: Decreased numbers of PBMCs and functional loss of NK cells obtained from cancer patients.**

PBMCs from healthy individuals and cancer patients were isolated from 30 ml of peripheral blood, and the number of cells counted using microscopy (n=14) **(A)**. PBMCs (2 x 10^5^ cells) were used to determine the percentages of CD16 (n=12), CD56 (n=12), CD3 (n=12), CD19 (n=9), CD14 (n=10), and CD11b (n=9) subsets within CD45+ immune cells using flow cytometric analysis (n=9-12) **(B)**. NK cells were isolated from PBMCs as described in the Materials and Methods section. Purified NK cells (1x10^6^ cells/ml) were left untreated or treated with IL-2 (1000 U/ml) for 18 hours before the supernatants were harvested and IFN-γ secretions were determined using single ELISA **(C)**. NK cells were isolated and treated as described in Fig. S1C and were added to ^51^Cr-labeled oral squamous cell carcinoma stem cells (OSCSCs) at various effector-to-target ratios. NK cell-mediated cytotoxicity was measured using a standard 4-hour ^51^Cr release assay against OSCSC. The lytic units (LU) 30/10^6^ cells were determined using the inverse number of NK cells required to lyse 30% of OSCSCs x 100 (n=9) **(D)**. NK cells were isolated and treated with IL-2 as described in Fig. S1C for 18 hours before the supernatants were harvested and ran with multiplex cytokine array kit to determine IFN-γ, IL-12p70, IL-6, TNF-α, IL-5, and IL-4 secretion **(E)**. Sera were obtained from the peripheral blood of healthy individuals (n=5) and cancer patients (n=8) as described in Supplementary Materials and Methods, and analyzed for the levels of cytokines, chemokines, and growth factors using a multiplex array kit **(F)**.

**Figure S2: OC-mediated activation of NK cells induced lower secretion of IFN-γ from cancer patients’ NK cells in comparison to healthy individuals.**

Purified NK cells (1 x 10^6^ cells/ml) from healthy individuals and cancer patients were treated with a combination of IL-2 (1000 U/ml) and anti-CD16 mAb (3 μg/ml) for 18 hours before they were treated with sAJ2 at a ratio of 1:2 (NK:sAJ2). The supernatants were then harvested from the co-cultures on days 6, 9, 12, and 15, and IFN-γ secretions were determined using single ELISA (n=8) **(A)**. Monocytes were purified from healthy individuals’ PBMCs and were then cultured in alpha-MEM media supplemented with M-CSF (25 ng/ml) and RANKL (25 ng/ml) for 21 days to generate OCs. Purified NK cells (1 x 10^6^ cells/ml) from the healthy individuals and cancer patients were treated with a combination of IL-2 (1000 U/ml) and anti-CD16 mAb (3 μg/ml) for 18 hours before they were co-cultured with sAJ2 and OCs at a ratio of 1:2:4 (OCs:NK:sAJ2). The supernatants were then harvested from the co-cultures on days 6, 9, 12, and 15, and IFN-γ secretions were determined using single ELISA (n=8) **(B)**.

**Figure S3: Decreased expansion and function of T cells from cancer patients with and without OC-mediated activation when compared to those from healthy individuals.**

OCs were generated as described in Fig. S2B. Purified T cells (1 x 10^6^ cells/ml) from healthy individuals and cancer patients were treated with a combination of IL-2 (100 U/ml) and anti-CD3 (1 µg/ml) and anti-CD28 (3 μg/ml) for 18 hours before they were treated with sAJ2 with and without OCs at a ratio of 1:2:4 (OCs:T:sAJ2). Cells were counted using microscopy on days 6, 9, 12, and 15 of the co-cultures and the cumulative cell counts of lymphocytes from day 0-day 15 were determined (n=4) **(A)**. Purified T cells from the healthy individuals and cancer patients were treated and cultured with OCs as described in Fig. S3A. The supernatants were harvested on days 6, 9, 12, and 15 of the co-cultures, and levels of IFN-γ were measured using single ELISA; the cumulative amounts of IFN-γ detected from day 0-day 15 is shown in the figure (n=4) **(B)** and the corresponding amount were adjusted based on 1 million cell counts (n=4) **(C)**. Purified T cells from healthy individuals and cancer patients were treated with a combination of IL-2 (100 U/ml) and anti-CD3 (1 µg/ml) and anti-CD28 (3 μg/ml) for 18 hours before they were treated with sAJ2 at a ratio of 1:2 (T:sAJ2). The supernatants were then harvested on days 6, 9, 12, and 15 of the co-cultures, and levels of IFN-γ were determined using single ELISA (n=4) **(D)**. Purified T cells from the healthy individuals and cancer patients were treated and cultured with OCs as described in Fig. S3A. The supernatants were harvested on days 6, 9, 12, and 15 of the co-cultures, and IFN-γ secretions were determined using single ELISA (n=4) **(E)**. Freshly purified NK cells from the healthy individuals were treated and cultured as described in Figs. S2A and S2B. Purified T cells from the healthy individuals were treated and cultured as described in Fig. S3A. Cells were then counted using microscopy on days 6, 9, 12, and 15 of the co-cultures and the cumulative lymphocyte counts from day 0-day 15 were determined (n=4) **(F)**.

**Figure S4: OC-mediated activation induced higher secretion of cytokines and chemokines from NK cells when compared to T cells.**

Freshly purified NK cells from the healthy individuals were treated and co-cultured as described in Fig. S2B. On day 12 of the co-culture; NK and NK-expanded CD8+ T cells were isolated from the expanded NK cells using the corresponding isolation kits. NK cells were treated with a combination of IL-2 (1000 U/ml) and anti-CD16 mAb (3 μg/ml) and, CD8+ T cells were treated with IL-2 (100 U/ml) and anti-CD3 (1 µg/ml)/CD28 mAb (3 μg/ml) for 18 hours. The supernatants were then harvested and were used to determine the levels of cytokines, chemokines, and growth factors using multiplex array kits. The amounts of all tested factors were adjusted based on 1 million cell counts and, the ratios of secretion between NK and CD8+ T cells (NK/CD8+ T cells) were determined and fold increase in the secreted levels for NK cells were determined **(A)**. Freshly purified NK cells from the healthy individuals were treated and co-cultured as described in Fig. S2B. Freshly purified T cells from the healthy individuals were treated and co-cultured with OCs as described in Fig. S3A. The supernatants were then harvested on day 6 of co-culture, and the levels of cytokines, chemokines, and growth factors were measured using multiplex array kits. Ratios of secretion between NK and T cells (NK/ T cells) were determined and fold increase in the secreted levels for NK cells were determined. **(B)**. The secreted levels shown in Fig. S4B were adjusted based on 1 million cell counts and, the ratios of secretion between NK and T cells (NK/ T cells) were determined and fold increase in the secreted levels for NK cells were determined **(C)**. Freshly purified NK cells from the healthy individuals were treated and co-cultured with OCs as described in Fig. S2B. In a separate culture, freshly isolated CD8+ T cells purified from healthy individuals were treated with IL-2 (100 U/ml) and anti-CD3 (1 µg/ml)/CD28 mAb (3 μg/ml) for 18 hours before they were cultured with OCs at a ratio of 1:2:4 (OCs:CD8+T:sAJ2). On day 12, CD8+ T cells were isolated from OC-expanded NK cells. CD8+ T cells isolated from OC-expanded NK cells and those from OC-expanded CD8+ T cells were further treated with IL-2 (100 U/ml) and anti-CD3 (1 µg/ml)/CD28 mAb (3 μg/ml) and after 18 hours of incubation, the supernatants were harvested from both CD8+ T cells cultures, and the levels of cytokines, chemokines, and growth factors were measured using multiplex array kits. Ratios of secretion between CD8+ T cells isolated from OC-expanded NK cells and OC-expanded CD8+ T cells were determined and fold increase in the secreted levels for CD8+ T cells isolated from OC-expanded NK cells were determined **(D)**. One of three representative experiments is shown in figure S4.

**Figure S5: OCs from Cancer patients had lower ability to expand autologous CD8+ T cells both in NK cells and T cells co-cultures in comparison to those from healthy individuals.**

Freshly purified NK cells (1 x 10^6^ cells/ml) from healthy individuals and cancer patients were treated with the combination of IL-2 (1000 U/ml) and anti-CD16mAb (3 µg/ml) for 18 hours. NK cells from healthy individuals and cancer patients were co-cultured with their respective autologous OCs in the presence of sAJ2 at a ratio of 1:2:4 (OCs:NK:sAJ2). Purified T cells (1 x 10^6^ cells/ml) from healthy individuals and cancer patients were treated with the combination of IL-2 (100 U/ml) and anti-CD3 (1 µg/ml)/CD28mAb (3 µg/ml) for 18 hours and then co-cultured with their respective autologous OCs. On day 9 of co-culture, surface expression of CD4 and CD8 were analyzed using flow cytometry, and the percentages of CD4+ and CD8+ T cells within CD3+ T cells were determined.

**Figure S6**: **OC-expanded NK cell immunotherapy increased cytokine secretion in the sera of hu-BLT mice.**

Reconstituted hu-BLT mice were injected with 1 x 10^6^ human OSCSCs into the floor of the mouth. One to two weeks later, hu-BLT mice were intravenously injected with 1.5 x 10^6^ OC-expanded NK cells. Disease progression was monitored for another 3-4 weeks, after which mice were sacrificed. Peripheral blood was collected in heparin-free vials post-mortem by cardiac puncture, and serum samples were harvested as described in Supplementary Materials and Methods, and analyzed for IFN-γ **(A)**, IL-6 **(B)**, ITAC **(C)**, IL-8 **(D),** and GM-CSF **(E)** secretions using multiplex arrays. One of the three representative experiments is shown in figure S6.

**Figure S7: NK cells preferentially lyse CD4+ T cells over CD8+ T cells.**

Freshly purified CD4+ T and CD8+ T cells from healthy individuals were treated with anti-CD3 (1 µg/ml) and IL-2 (100 U/ml) for 18 hours. Freshly purified NK cells from healthy individuals were treated with a combination of IL-2 (1000 U/ml) and anti-CD16mAb (3 µg/ml) for 18 hours before they were used to determine NK cell-mediated cytotoxicity against CD4+ and CD8+ T cells using a TVA assay. The lytic units (LU) 30/10^7^ cells were determined using inverse number of NK cells required to lyse 30% of target cells x 100 (n=3).

**Figure S1**


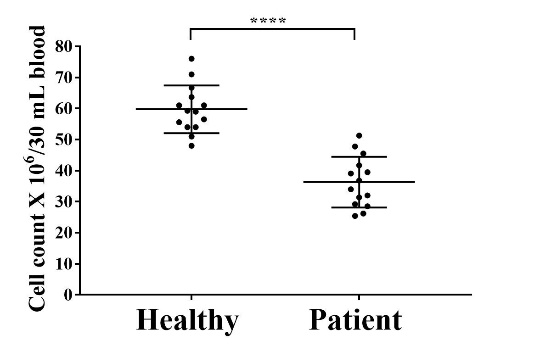
**Fig. S1A**

**Fig. S1B**

**
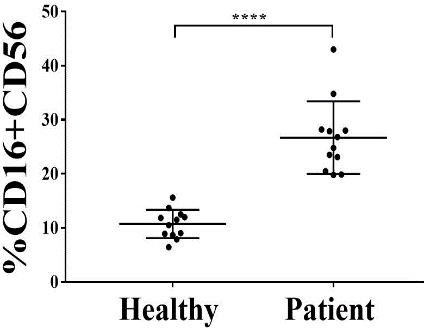

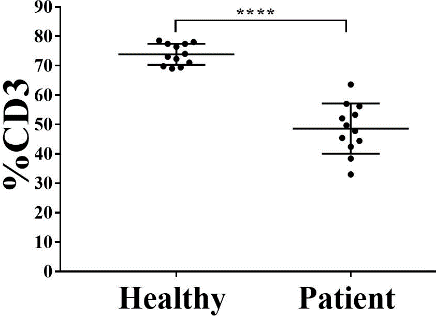

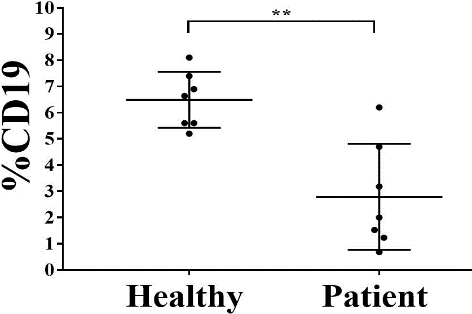
**

**
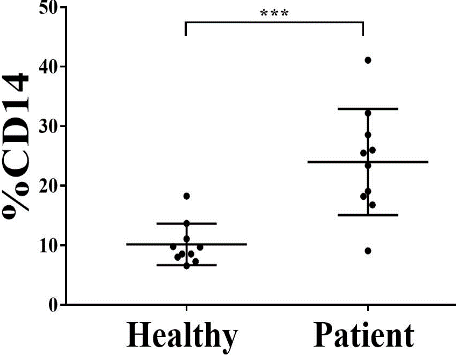

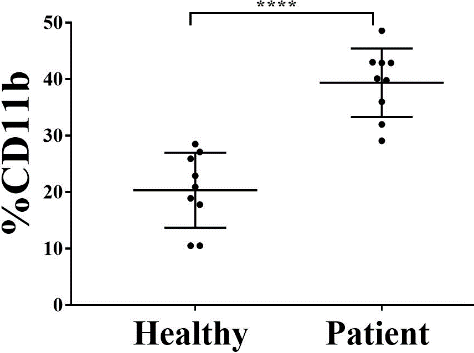
**

**Fig. S1C Fig. S1D**

**
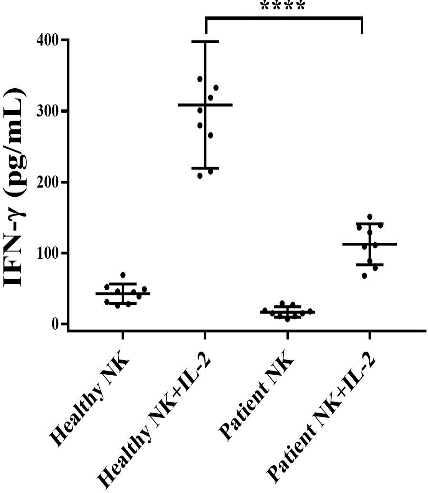

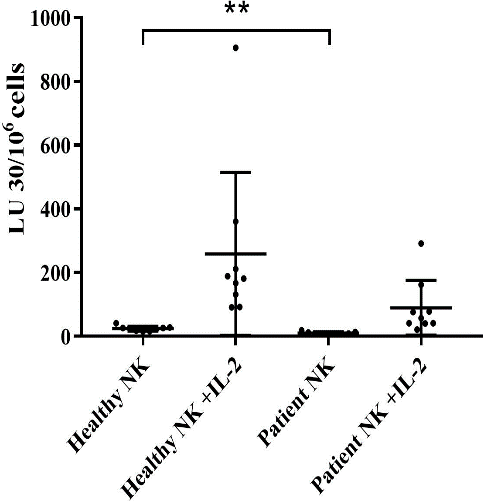
**

**Fig. S1E**


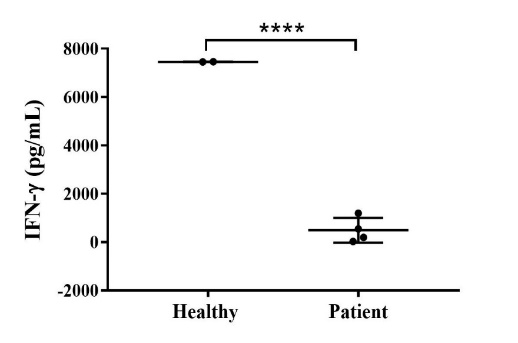

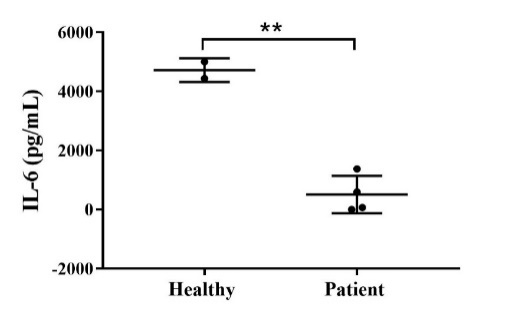

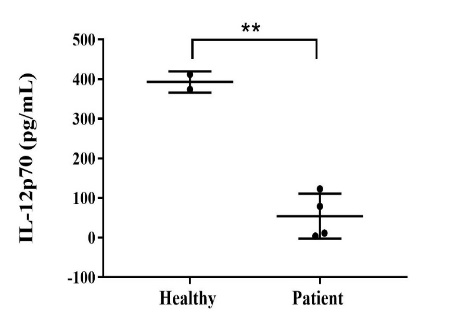


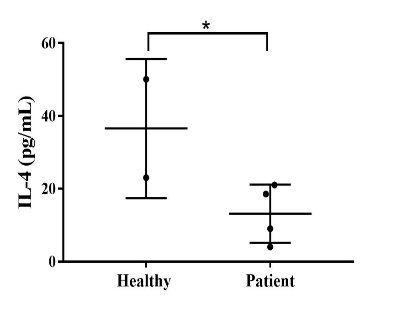

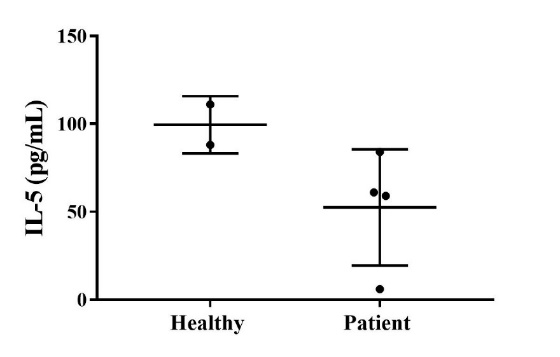

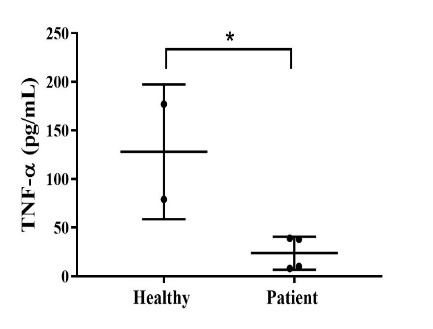


**Fig. S1F**


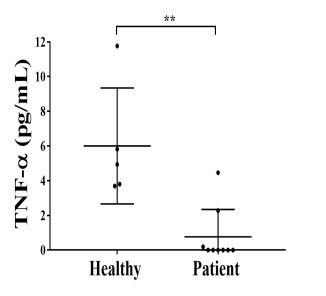

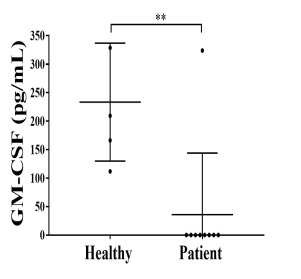

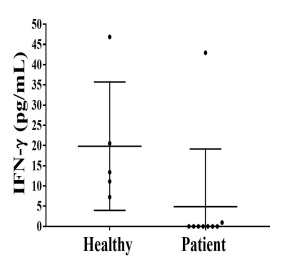

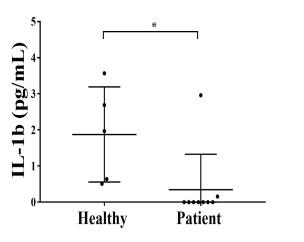

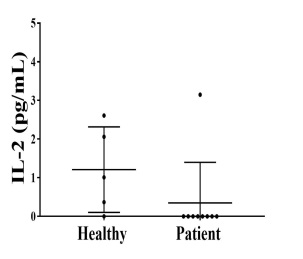

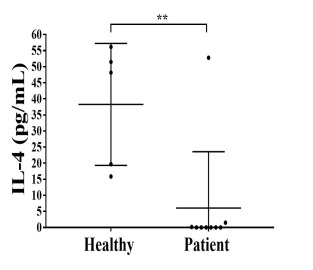

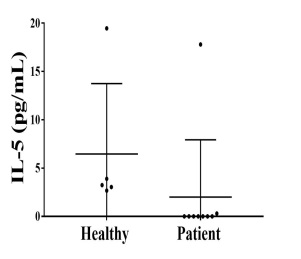

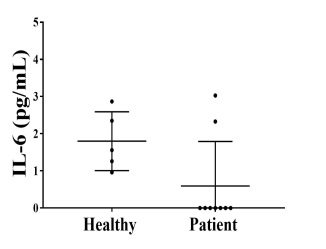

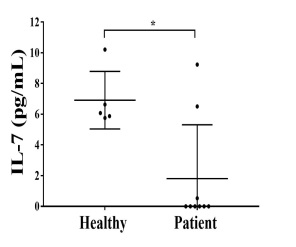

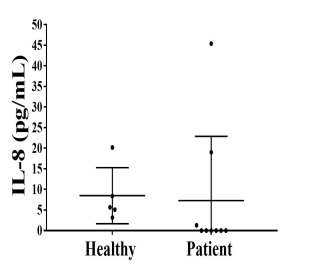

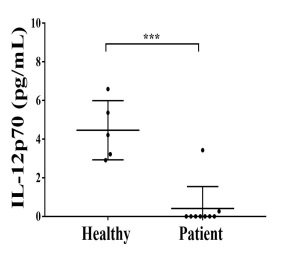

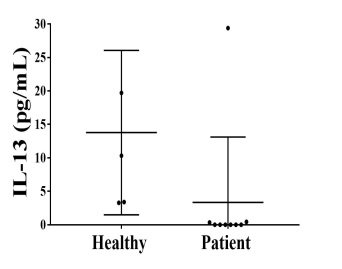

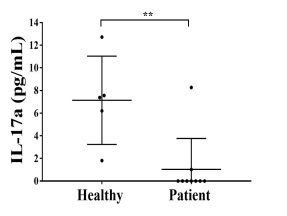

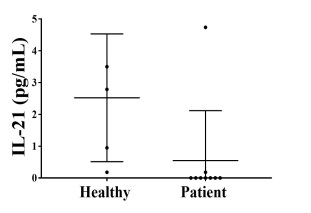

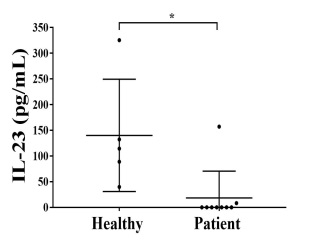

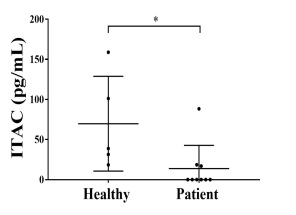

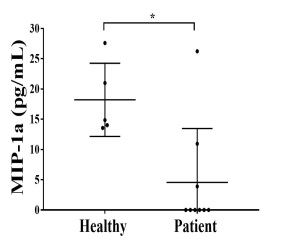

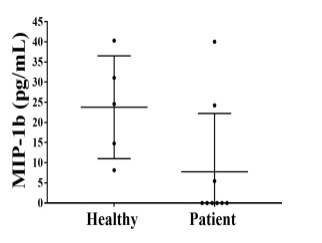

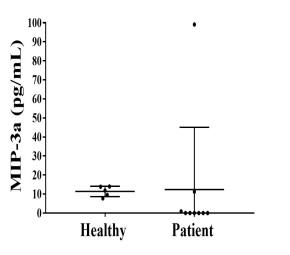

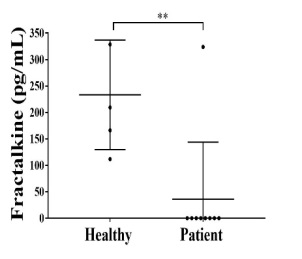

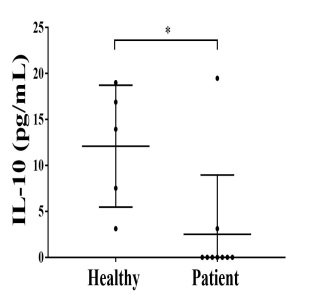


**Figure S2**

**Fig. S2A Fig. S2B**

**
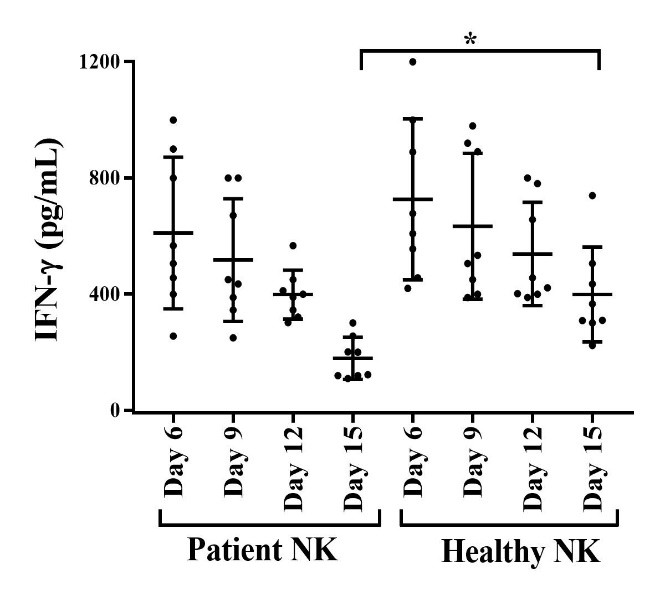

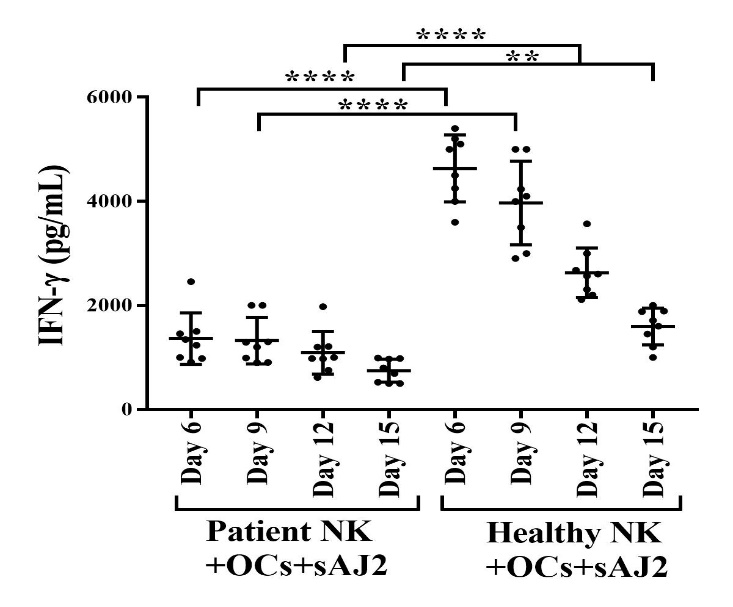
**

**Figure S3**

**Fig. S3A Fig. S3B Fig. S3C**


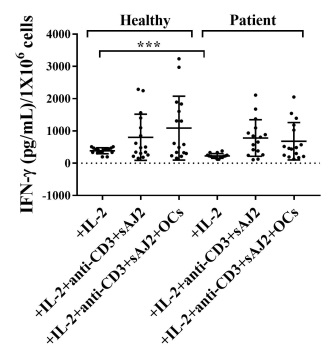
**
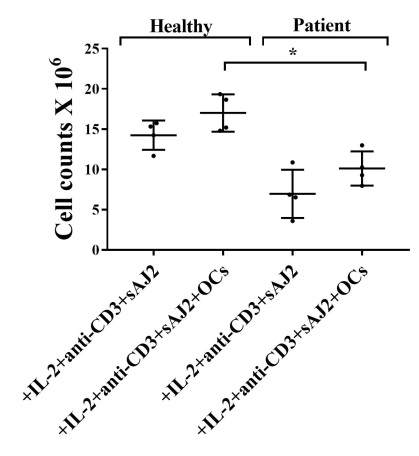
**


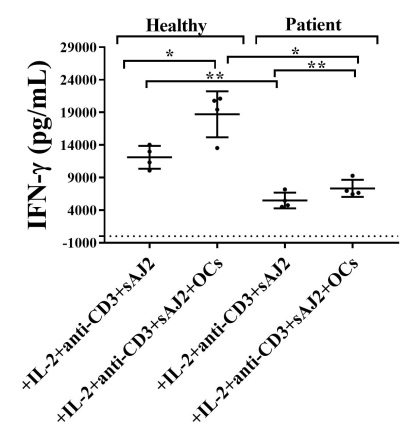


**Fig. S3D Fig. S3E**

**
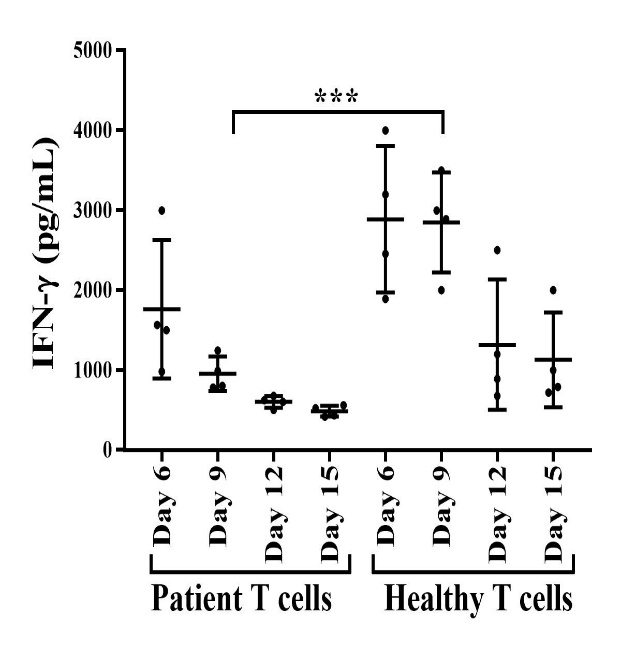

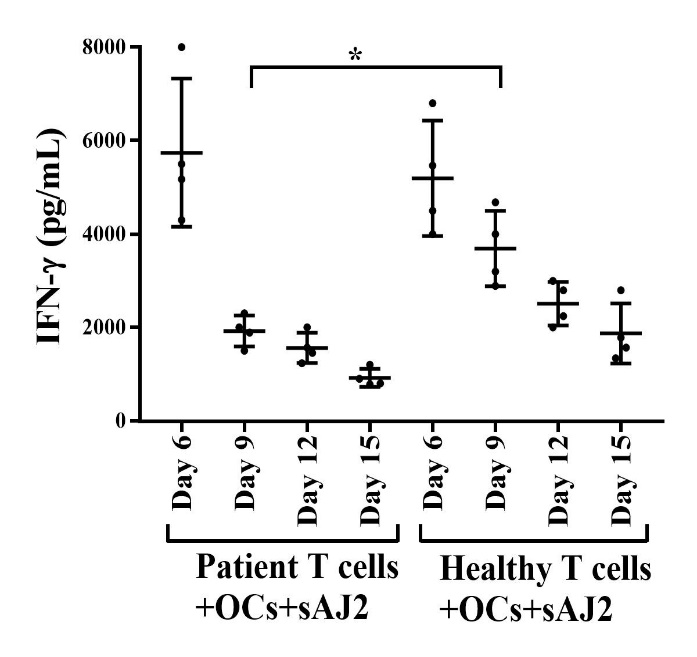
**

**Fig. S3F**

**
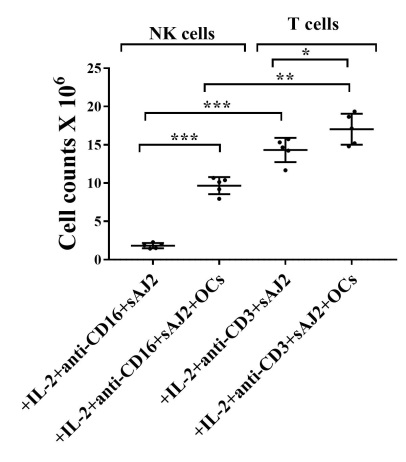
**

**Figure S4**

**Fig. S4A**


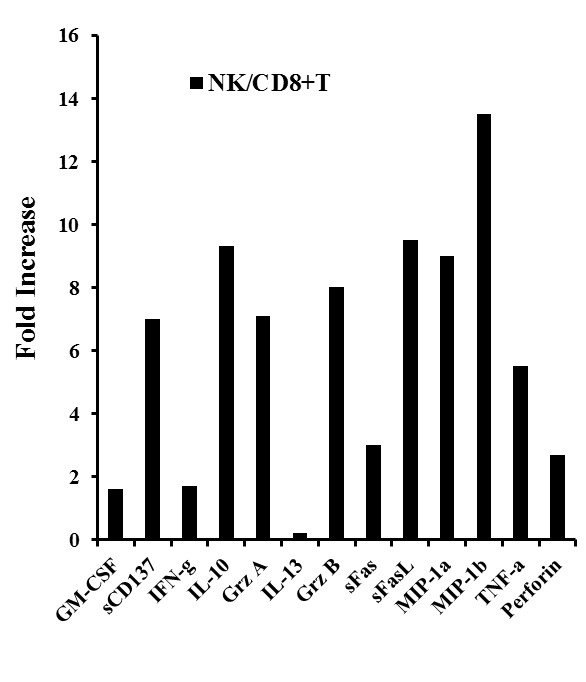


**Fig. S4B Fig. S4C**


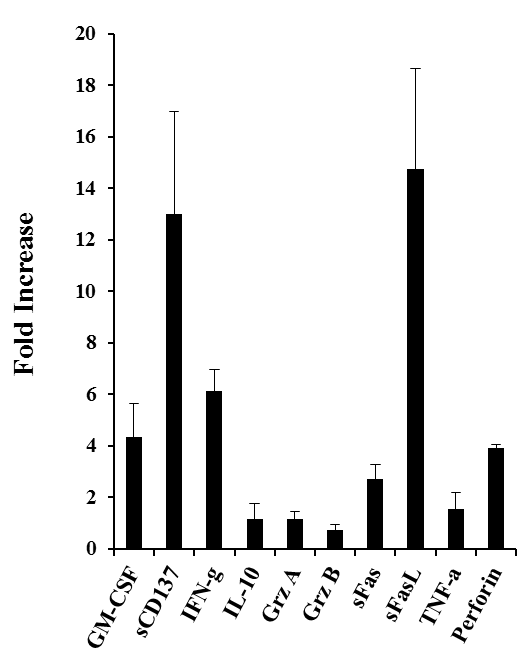

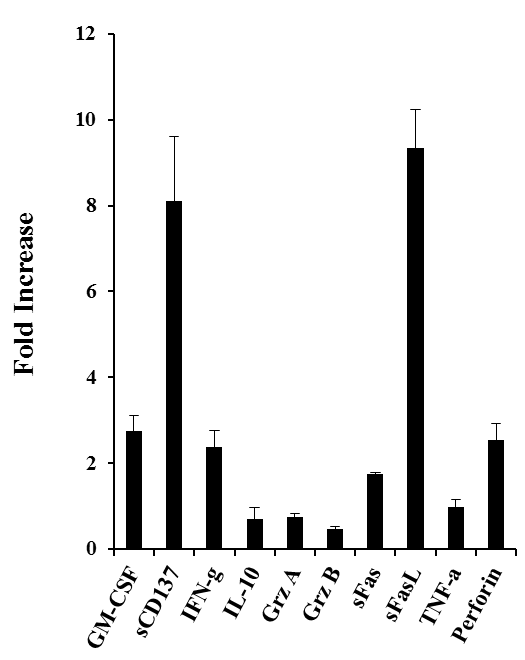


**Fig. S4D**
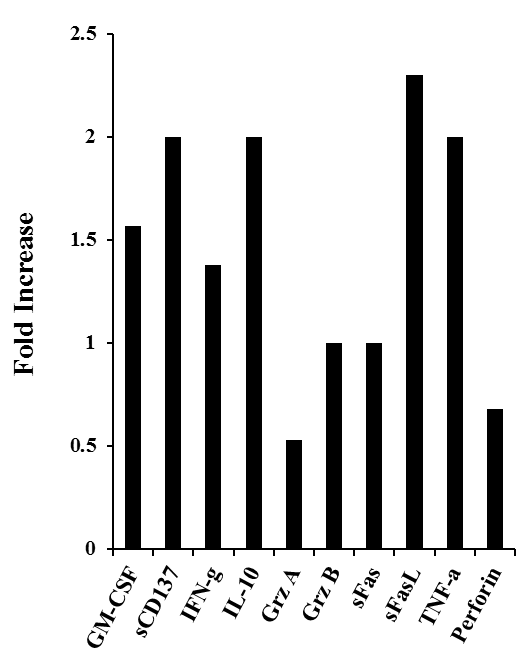


**Figure S5**


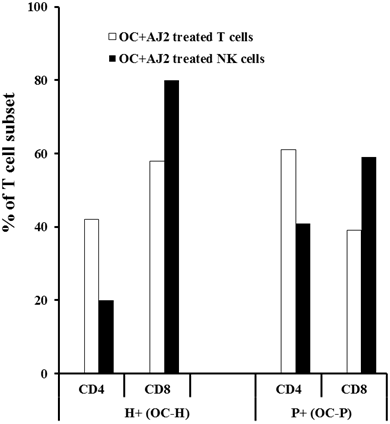


**Figure S6**

**Fig. S6A Fig. S6B Fig. S6C Fig. S6D Fig. S6E**


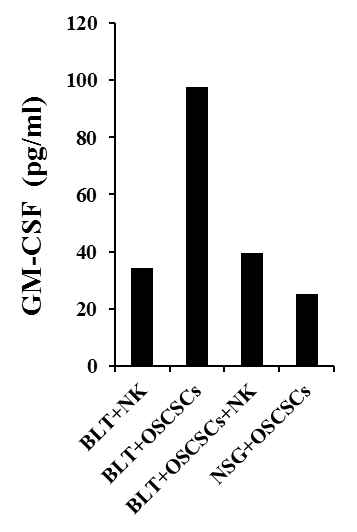

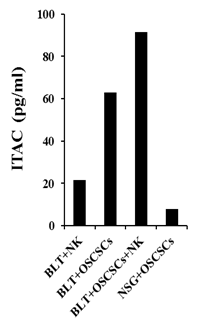

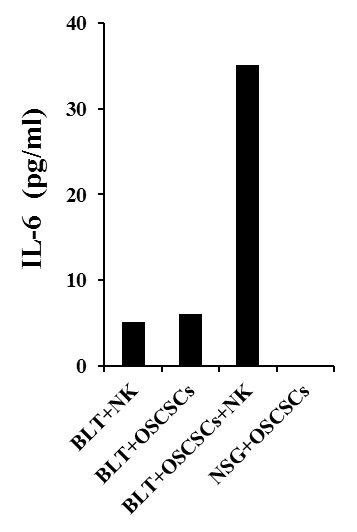

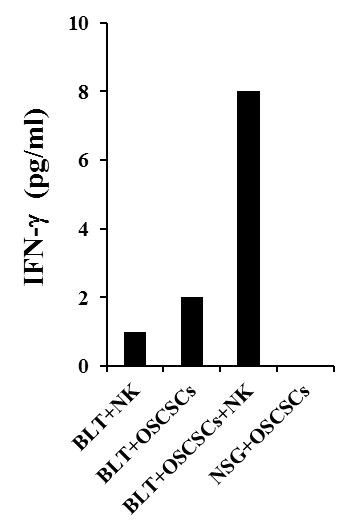


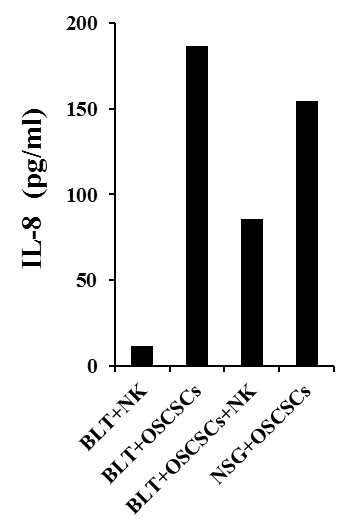


**Figure S7**


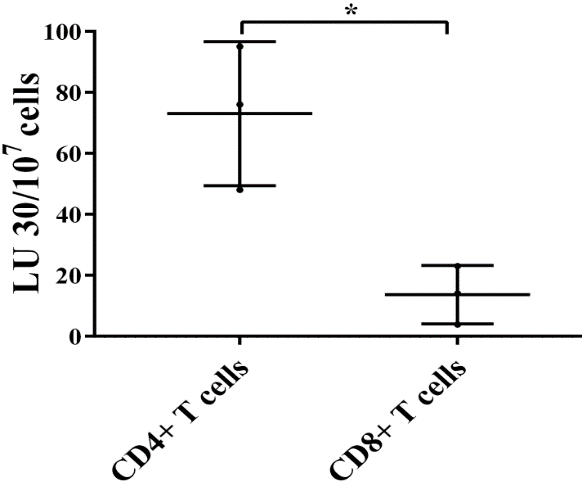

Supplement: Supplementary file 1 — Supplementary information. [file 41598_2020_76702_MOESM1_ESM.docx]
